# Augmenting MNK1/2 activation by c-FMS proteolysis promotes osteoclastogenesis and arthritic bone erosion

**DOI:** 10.1038/s41413-021-00162-0

**Published:** 2021-10-20

**Authors:** Se Hwan Mun, Seyeon Bae, Steven Zeng, Brian Oh, Carmen Chai, Matthew Jundong Kim, Haemin Kim, George Kalliolias, Chitra Lekha Dahia, Younseo Oh, Tae-Hwan Kim, Jong Dae Ji, Kyung-Hyun Park-Min

**Affiliations:** 1 Arthritis and Tissue Degeneration Program, David Z. Rosensweig Genomics Research Center, New York, NY USA; 2 Tissue Engineering, Regeneration and Repair, Hospital for Special Surgery, New York, NY USA; 3 Department of Cell and Developmental Biology, Weill Cornell Medical College, New York, NY USA; 4 BCMB allied program, Weill Cornell Graduate School of Medical Sciences, New York, NY USA; 5 Hanyang University Institute for Rheumatology Research, Seoul, Korea; 6 Department of Rheumatology, Hanyang University Hospital for Rheumatic Diseases, Seoul, Korea; 7 Rheumatology, College of Medicine, Korea University, Seoul, Korea; 8 Department of Medicine, Weill Cornell Medical College, New York, NY USA

**Keywords:** Bone, Bone quality and biomechanics

## Abstract

Osteoclasts are bone-resorbing cells that play an essential role in homeostatic bone remodeling and pathological bone erosion. Macrophage colony stimulating factor (M-CSF) is abundant in rheumatoid arthritis (RA). However, the role of M-CSF in arthritic bone erosion is not completely understood. Here, we show that M-CSF can promote osteoclastogenesis by triggering the proteolysis of c-FMS, a receptor for M-CSF, leading to the generation of FMS intracellular domain (FICD) fragments. Increased levels of FICD fragments positively regulated osteoclastogenesis but had no effect on inflammatory responses. Moreover, myeloid cell-specific FICD expression in mice resulted in significantly increased osteoclast-mediated bone resorption in an inflammatory arthritis model. The FICD formed a complex with DAP5, and the FICD/DAP5 axis promoted osteoclast differentiation by activating the MNK1/2/EIF4E pathway and enhancing NFATc1 protein expression. Moreover, targeting the MNK1/2 pathway diminished arthritic bone erosion. These results identified a novel role of c-FMS proteolysis in osteoclastogenesis and the pathogenesis of arthritic bone erosion.

## Introduction

Rheumatoid arthritis (RA) is a chronic inflammatory and autoimmune disorder^1^. Bone erosion is one of the key clinical features of RA and is closely linked to impaired mobility in patients with RA^2^. However, the underlying mechanisms of arthritic bone erosion by osteoclasts have not been fully determined^3^. In addition to inflammatory cytokines such as tumor necrosis factor-alpha (TNFα), macrophage colony-stimulating factor (M-CSF) and its receptor c-FMS have also been implicated in the pathogenesis of RA and arthritic bone erosion^4^. In patients with RA, the level of M-CSF is increased in the serum and synovial fluid^5^, and inhibiting c-FMS activation attenuates the progression of joint inflammation and bone erosion in animal models of arthritis^6^. Despite the importance of M-CSF in the differentiation of myeloid cells^7^, very little is known about the molecular mechanism underlying the role of M-CSF/c-FMS in arthritic bone erosion.

Ectodomain shedding is critical for the function of various membrane proteins. Many cell surface proteins, such as Notch, undergo proteolysis via regulated intracellular proteolysis (RIP) to generate functional small fragments of membrane-anchored proteins^8^. This process is mediated by a disintegrin and metalloprotease (ADAM) and γ-secretase. c-FMS also undergoes proteolysis by tumor necrosis α converting enzyme (TACE) and γ-secretase, generating small fragments that degrade once cells are exposed to an inflammatory stimulus^9,10^. c-FMS proteolysis is believed to cause its breakdown and termination of its functions^10,11^. Due to the importance of c-FMS in myeloid cells, the functions and downstream signaling pathways of c-FMS and its interacting ligands have been studied intensively. Despite this research, the role of c-FMS proteolysis remains largely unknown.

Osteoclasts are bone-resorbing cells derived from myeloid lineage cells that are responsible for arthritic bone erosion^12–14^. There are many cellular sensors and effector proteins that play roles in the generation and ultimate function of osteoclasts. Of those factors, M-CSF and receptor activator of NF-κB ligand (RANKL) are essential factors in the function and differentiation of monocytes and osteoclasts^13–16^. M-CSF signaling induces the expression of receptor activator of NF-κB (RANK), a receptor for RANKL, and RANKL then induces the expression of nuclear factor of activated T cells, cytoplasmic 1 (NFATc1), a master regulator of osteoclastogenesis, to initiate the osteoclast differentiation program^12^.

Transcription factor networks involved in NFATc1 mRNA expression have been well characterized, but the regulatory mechanisms of NFATc1 protein expression remain unclear. mRNA translation is tightly controlled at multiple levels, and alterations in protein synthesis can lead to disease or cellular apoptosis^17^. The initiation of protein synthesis is a rate-limiting step. This step is facilitated by eukaryotic initiation factor (eIF)4F, which binds to the 5′ cap, m^7^GTP, of mRNAs, to recruit the mRNA to the ribosome. eIF4F is a multisubunit protein complex composed of eIF4A, eIF4E, and eIF4G. eIF4G is recruited to mRNA and forms the 43 S preinitiation complex, which consists of three protein family members: eIF4GI (eIF4G1), eIF4GII, and death-associated protein 5 (DAP5, also known as eIF4G2). In contrast to the well-known function of eIF4G1, the role of DAP5 in protein translation is controversial. A recent study showed that DAP5 could form inactive complexes and suppress protein translation^18^. Another study revealed that the DAP5 complex promoted alternative translation of specific subsets of mRNAs^19^. Furthermore, the full function of the DAP5 complex has not been defined, and the role of DAP5 in myeloid cells is unknown.

We showed that ligand engagement of c-FMS generated FMS intracellular domain (FICD) fragments in both human and murine macrophages via proteolysis. Here, we showed that increased FICD protein levels in arthritic synovial macrophages promoted osteoclast differentiation and arthritic bone erosion. Using gain-of-function and loss-of-function studies, we demonstrated that FICD fragments enhanced osteoclast differentiation and activity. Furthermore, myeloid-specific FICD transgenic mice exhibited an osteoporotic phenotype with increased osteoclasts and the promotion of arthritic bone erosion compared with those of control mice. This positive role of the FICD in osteoclasts was mediated by accelerating MAP kinase-interacting serine/threonine-protein kinase 1 (MNK1/2) activation and NFATc1 expression by binding to DAP5. Overall, our findings elucidate the molecular mechanisms of c-FMS proteolysis in osteoclasts and reveal how c-FMS proteolysis accelerates RANKL-induced osteoclast differentiation and arthritic bone erosion. Our results also provide a strong basis for a new therapeutic target for pathological bone resorption in RA.

## Results

### Synovial CD14^+^ cells show a distinct c-FMS expression pattern

**Fig. 1:**
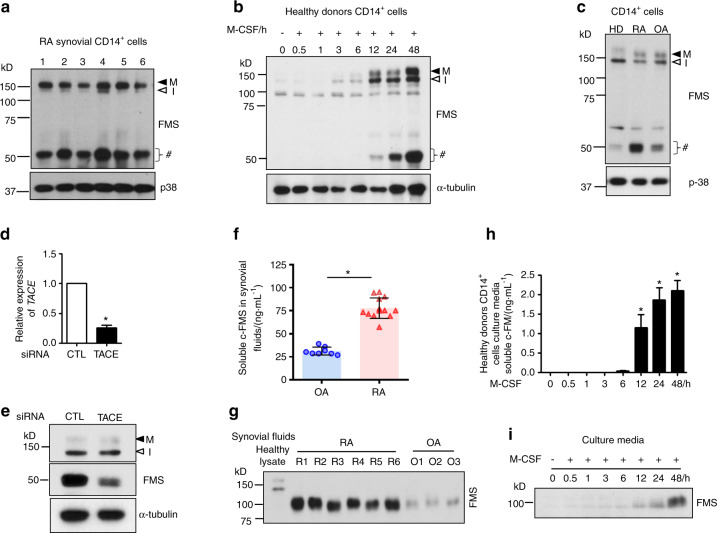
The detection of small fragments of c-FMS and soluble c-FMS. **a** Immunoblot analysis of rheumatoid arthritis (RA) synovial CD14^+^ cells with antibodies against the C-terminus of c-FMS. **b** Human CD14^+^ cells from healthy donors were cultured with M-CSF for the indicated times. Immunoblot analysis of whole cell lysates with antibodies against the C-terminus of c-FMS. **c** Immunoblot analysis of whole cell lysates from CD14^+^ cells from healthy donors cultured with M-CSF for 1 day (HD) and synovial CD14^+^ cells from patients with RA or osteoarthritis (OA). **d**, **e** Human CD14^+^ cells were nucleofected with control (CTL) or TACE siRNAs and then cultured with M-CSF. **d** Knockdown (KD) efficiency. TACE mRNA was measured by qPCR and normalized to HPRT. **e** Immunoblot analysis of KD cells using an anti-c-FMS antibody. **f**, **g** Soluble c-FMS levels in synovial fluids from patients with rheumatoid arthritis (RA, *n* = 13) and osteoarthritis (OA, *n* = 8) were measured by ELISA (**f**) and immunoblot analysis with antibodies against N-terminal c-FMS (**g**). **h**, **i** Human CD14^+^ cells were cultured with M-CSF. Soluble c-FMS in the culture media was measured by ELISA (**h**) and immunoblot analysis with anti-c-FMS antibodies (**i**). All data are shown as the mean ± SEM. *P < 0.05 by unpaired *t*-test (**d**, **f**) or one-way ANOVA with *a post hoc Tukey* test (**h**). The data represent at least 3 independent donors. M; a mature c-FMS, I; an immature c-FMS, #; small fragments.

M-CSF/c-FMS signaling is implicated in the pathogenesis of RA. Consistent with a previous report showing increased M-CSF expression in RA synovial fluids^5^, M-CSF levels were significantly higher in RA synovial fluids than in osteoarthritis (OA) synovial fluids (Supplementary Fig. 1a). We also measured the expression of cell-associated c-FMS in synovial CD14^+^ cells from RA patients. A c-FMS antibody against the C-terminal region of the receptor detected mature, glycosylated c-FMS (150 kD, M) and immature, unglycosylated c-FMS (130 kD, I), as expected. Intriguingly, we also detected small fragments of ~50 kD in synovial CD14^+^ cells using anti-c-FMS antibodies (Fig. 1a). We next examined whether CD14^+^ cells from healthy donors expressed small fragments. Immunoblot analysis of freshly isolated CD14^+^ cells showed low levels of mature and immature c-FMS, but small fragments were hardly detectable (Fig. 1b). After fresh CD14^+^ cells were cultured with M-CSF, the levels of mature and immature c-FMS and small fragments increased in a time-dependent manner (Fig. 1b). When we compared c-FMS expression between freshly isolated RA synovial CD14^+^ cells, M-CSF-cultured CD14^+^ cells from healthy donors, and OA synovial CD14^+^ cells, we found higher levels of small fragments in RA synovial CD14^+^ cells, while the levels of mature and immature c-FMS were comparable (Fig. 1c). To examine whether the observed 50-kD bands originated from c-FMS, mass spectrometry analysis was performed on the 50-kD gel bands after immunoprecipitation with a c-FMS C-terminal antibody. Indeed, c-FMS was detected as the top ranked protein in the 50-kD gel by mass spectrometry (Supplementary Fig. 1b). To corroborate our findings, we examined whether commercially available anti-c-FMS antibodies could detect the small fragments. The 50-kD small fragments were detected by all antibodies against the C-terminal region of c-FMS (Supplementary Fig. 1c–e). However, these fragments were not detected by antibodies against the N-terminal region of c-FMS (Supplementary Fig. 1f, g). These results suggest that the 50-kD bands contained C-terminal regions of c-FMS. We named these 50 kD bands c-FMS intracellular domain (FICD) fragments.

Among peripheral blood mononuclear cells (PBMCs), only CD14^+^ cells are able to differentiate into osteoclasts^20^. To examine whether FICD fragments were generated by c-FMS proteolysis, TACE expression was knocked down using short interfering RNAs (siRNAs) in freshly isolated CD14^+^ cells, and the cells were cultured with M-CSF. TACE was decreased by 75% by TACE siRNA compared with control siRNA (Fig. 1d). As a result, TACE knockdown diminished the generation of FICD fragments in response to M-CSF stimulation (Fig. 1e). Accordingly, treatment with the MMP inhibitor BB94 also suppressed the generation of FICD fragments (Supplementary Fig. 1h) and inhibited ectodomain shedding of c-FMS (Supplementary Fig. 1i). These results suggested that TACE cleavage was required for the generation of FICD fragments. To examine whether the increase in FICD fragments in RA synovial CD14^+^ cells correlated with the shedding of c-FMS, we measured the level of soluble c-FMS in RA and OA synovial fluids. Soluble c-FMS was detectable by ELISA and immunoblot analysis, and the level of soluble c-FMS was higher in RA synovial fluid than in OA synovial fluid (Fig. 1f). In addition, the soluble c-FMS in synovial fluids had a smaller molecular weight than full-length c-FMS (Fig. 1g), suggesting that c-FMS proteolysis may be active in the RA synovium. Accordingly, soluble c-FMS was not detected in freshly isolated CD14^+^ cells from healthy donors, but soluble c-FMS secretion in the media gradually increased during culture with M-CSF (Fig. 1h, i). However, other serological markers, including C-reactive protein (CRP), the erythrocyte sedimentation rate (ESR), and anti-cyclic citrullinated peptide (CCP) antibodies, were not associated with FICD expression in RA synovial CD14^+^ cells (Supplementary Fig. 2a–c).

### M-CSF mediates the generation of FICD fragments

**Fig. 2:**
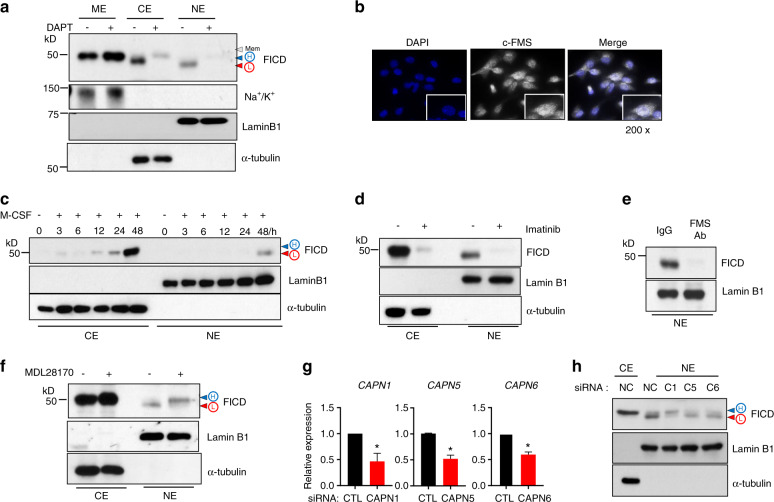
Calpain 1 cleaves FICD fragments. **a** Human CD14^+^ cells were cultured with M-CSF (20 ng·mL^−1^) for 8 h to induce early signals, and then DAPT (10 μmol·L^−1^) was added. The cells were cultured for additional 2 days. Protein expression of c-FMS, the Na^+^/K^+^pump, Lamin B1, and α-tubulin were determined by immunoblot analysis. ME membrane extracts, CE cytoplasmic extracts, NE nuclear extracts. **b** Immunocytochemical analysis of DAPI and c-FMS. The right panel shows a merged image. Scale: 200×. **c** Cells were starved for 3h and then stimulated with M-CSF for the indicated times. **d**, **e** Cells were treated with imatinib (0.3 μmol·L^−1^, **d** or a c-FMS-blocking antibody (5 μg·mL^−1^, **e** prior to the addition of M-CSF. Protein expression of the FICD was measured by immunoblot analysis. Lamin B1 and α-tubulin were used as controls for the nuclear and cytoplasmic fractions, respectively. **f** Human CD14^+^ cells were cultured with M-CSF (20 ng·mL^−1^) for 8h to induce early signals, and then MDL 28170 (5 μmol·L^−^^1^) was added. The cells were cultured for additional 2 days. Immunoblot analysis with anti-c-FMS, Lamin B1, and α-tubulin antibodies. **g**, **h** Calpain 1, 5, and 6 were knocked down with siRNAs. The cells were cultured with M-CSF for 12 h. **g** Efficiency of Calpain 1, 5, and 6 knockdown. **h** Immunoblot analysis of c-FMS, α-tubulin, and Lamin B1. All data are shown as the mean ± SEM. *P < 0.05 by two-tailed, unpaired *t*-test (**g**). Representative results from at least three independent experiments are shown.

Consistent with previous reports^13,17^, c-FMS proteolysis was initiated by TACE (Fig. 1). We reasoned that FICD generation followed a conventional RIP process through ADAM family proteins and ɣ-secretase^8^. It has been shown that c-FMS has cleavage sites for ɣ-secretase in transmembrane regions^10^. Macrophages were treated with DAPT, a small molecule inhibitor of ɣ-secretase^21^. Then, the cells were lysed and fractionated into three cellular compartments: membrane, cytoplasmic, and nuclear. When we inhibited γ-secretase with DAPT, FICD remained in the membrane, and membrane-bound FICD accumulated (Fig. 2a). The membrane-bound form of the FICD had the highest molecular weight (mem), followed by the slightly smaller cytoplasmic FICD, which was denoted high molecular mass FICD (H-FICD). Both forms were larger than nuclear FICD, which was denoted L-FICD (low molecular mass FICD). We found that the cytosolic and nuclear FICD fragments were suppressed by DAPT (Fig. 2a). To further confirm the cellular localization of c-FMS and FICD fragments, we performed immunocytochemistry using an antibody against the C-terminal region of c-FMS in human macrophages, and signals were detected by fluorescence analysis and confocal microscopy. Consistent with the immunoblot analysis results, positive signals for c-FMS were detected in the membrane (mature form and mem), Golgi (immature form), cytoplasm (H-FICD), and nucleus (L-FICD) (Fig. 2b, Supplementary Fig. 3a). Since c-FMS signaling is required for FICD generation, we also examined whether c-FMS signaling contributed to the cellular localization of FICD fragments. Both M-CSF and IL-34, which are ligands for c-FMS^22^, induced the generation of H-FICD and L-FICD and the cellular distribution of FICD fragments (Fig. 2c, Supplementary Fig. 3b, c). Macrophages were treated with imatinib mesylate, an inhibitor of c-FMS activity, or with a c-FMS blocking antibody, which suppressed not only FICD generation but also the levels of nuclear FICD fragments (Fig. 2d, e). Taken together, our results established that M-CSF/c-FMS signaling positively regulates the generation and cellular localization of FICD fragments in macrophages.

These results suggest that an additional protease may cleave H-FICD to form L-FICD. To identify the protease(s) responsible for the cleavage of H-FICD in an unbiased manner, we screened 53 protease inhibitors using a protease library. The best hits associated with the inhibition of L-FICD generation were MDL28170 and PD150606 (two calpain inhibitors), along with MMP inhibitors and γ-secretase inhibitors. Calpain is a family of calcium-dependent cytosolic cysteine proteases expressed ubiquitously in mammals and many other organisms^23^, and calpain-dependent cleavage contributes to the modulation of various cellular functions, such as apoptosis, proliferation and migration^24,25^. Calpain has been implicated in osteoclastogenesis and migration^26,27^, although the exact mechanisms and targets of calpain are unknown. We found that inhibiting calpain suppressed the generation of the FICD in a dose-dependent manner (Supplementary Fig. 4a, b). The inhibition of calpain by MDL28170 did not interfere with the nuclear translocation of the FICD but instead shifted the enrichment of L-FICD to H-FICD in the nucleus, suggesting that calpain cleavage may occur in the nucleus (Fig. 2f). Since calpain is activated by calcium, we examined whether calcium signaling could compensate for M-CSF signaling to generate FICD fragments. In the absence of c-FMS signaling, calcium signaling was able to promote the cleavage of L-FICD to H-FICD in the nucleus, and MDL29170 reversed the effect of calcium signaling on L-FICD processing (Supplementary Fig. 4c). Consistent with the previous results, treatment with MDL28170 decreased not only L-FICD but also osteoclast differentiation (Supplementary Fig. 4d). To further investigate which form of calpain cleaves FICD in the nucleus, we used siRNAs to knock down Calpain 1, 5, and 6, which are expressed in macrophages. Calpain 1, 5, and 6 were efficiently knocked down using siRNAs (Fig. 2g). Among them, calpain 1-knockdown (KD) cells were unable to process H-FICD to L-FICD, resulting in H-FICD accumulation in the nucleus in calpain 1-KD cells (Fig. 2h). Thus, our results reveal that calpain 1 plays a key role in the proteolysis of H-FICD to form L-FICD. To address the (patho)physiological importance of the FICD in inflammatory bone erosion, we examined the effect of MDL28170 on bone erosion in K/BxN serum transfer-induced arthritis. K/BxN serum was administered intraperitoneally on day 0 and day 2, and then MDL28170 was administered after disease onset (Supplementary Fig. 4e). The severity of arthritis was assessed by clinical score and ankle joint thickness, which were attenuated by MDL28170 treatment (Supplementary Fig. 4f, g). Treatment with a calpain inhibitor decreased the number of osteoclasts and attenuated bone erosion in the K/BxN serum-induced arthritis model (Supplementary Fig. 4h, i). Taken together, our results suggest that c-FMS proteolysis generates FICD though sequential proteolysis via TACE, ɣ-secretase, and calpain 1.

### Blocking c-FMS proteolysis suppresses RANKL-induced osteoclast formation and activity

**Fig. 3:**
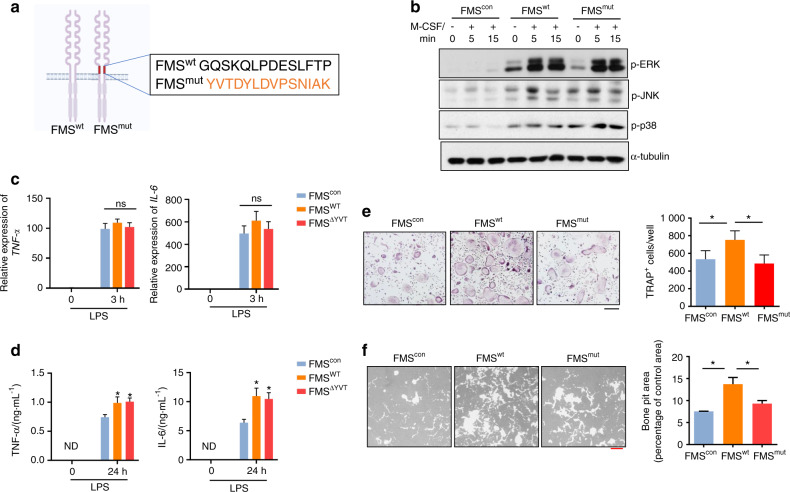
c-FMS proteolysis positively regulates osteoclastogenesis. **a** Schematic showing mutations in the TACE cleavage sites of c-FMS. TACE cleavage sites of c-FMS^10^ were replaced by the addition of 14 amino acids from insulin receptor sequences (FMS^mut^). **b** 293 T cells did not express c-FMS and were transduced with lentiviral particles encoding control, FMS^wt^ or FMS^mut^. The cells were then stimulated with M-CSF for the indicated times. Protein expression of phospho-ERK, phospho-JNK, phospho-p38, and α-tubulin was determined by immunoblot analysis. **c**–**f** BMDMs from c-FMS^f/+^ Mx1-Cre mice were transduced with lentivirus encoding control, wild-type FMS (FMS^wt^), or the TACE-uncleavable mutant FMS (FMS^mut^). The transduced BMDMs were stimulated with LPS (10 ng·mL^−1^) for 3 h (**c**) and 24 h (**d**). **c** The mRNA expression of TNFα and IL6 was measured by q-PCR. **d** TNFα and IL6 protein levels in the culture media were measured by Luminex multiplex cytokine assays. **e** Osteoclastogenesis assay. The left panel shows representative images of TRAP-stained cells. The right panel shows the percentage of TRAP-positive multinuclear cells (MNCs: more than three nuclei) per control (*n* = 6). Black scale bar: 100 μm. **f** Resorption pit assay. Bone resorption activity analysis of FMS^con^, FMS^wt^, or FMS^mut^ cells. The left panel shows representative images, and the right panel shows the percentage of the resorbed pit area per total area. Red scale bar: 200 μm. All data are shown as the mean ± SEM. ns not significant, ND not detected. *P < 0.05 by one-way ANOVA with *a post hoc Tukey* test (**c**–**f**). The data represent at least three experiments (**b**–**d**, **f**).

Given that FICD levels were increased in RA synovial CD14^+^ cells and that the administration of a calpain inhibitor suppressed both inflammation and bone erosion, we hypothesized that c-FMS proteolysis plays an important role in the functions of macrophages, including inflammatory responses and osteoclastogenesis. To test our hypothesis, we used noncleavable c-FMS mutants with mutated TACE cleavage sites (FMS^mut^, Fig. 3a) that could not produce FICD fragments^10^. 293 T cells that had no endogenous c-FMS expression were transduced with lentiviral particles encoding control, wild-type FMS (FMS^wt^), or FMS^mut^. Cell surface expression of both FMS^wt^ and FMS^mut^ was determined by flow cytometry, and FMS^mut^ was resistant to TPA-induced TACE-mediated shedding compared with FMS^wt^ (Supplementary Fig. 5a). Importantly, when the cells were stimulated with M-CSF, the activation of ERK, JNK, and p38 by FMS^mut^ was comparable to that of FMS^wt^ (Fig. 3b), suggesting that FMS^mut^ is a functional receptor. To minimize the effect of endogenous FICD, we used bone marrow-derived macrophages (BMDMs) from c-FMS inducible conditional haplodeficient mice (c-FMS ^f/+ΔMx1^) that were generated by crossing c-FMS floxed mice with Mx1 cre mice^28^, which expressed low levels of endogenous FICD (Supplementary Fig. 5b). FMS haplodeficient BMDMs from FMS^f/+^Mx1cre mice were transduced with lentiviral particles encoding control, FMS^wt^ or FMS^mut^. As expected, FICD generation in both FMS^mut^-transduced FMS haplodeficient BMDMs and 293 T cells was diminished compared to that in FMS^wt-^transduced cells (Supplementary Fig. 5c, d). To test the effect of c-FMS proteolysis on inflammatory responses, control, FMS^wt^-, and FMS^mut^-transduced cells were stimulated with LPS, a Toll-like receptor 4 (TLR4) agonist, and we measured the expression of proinflammatory cytokines such as TNFα and IL6. The mRNA expression of TNFα and IL6 was induced by LPS and was comparable among the groups (Fig. 3c). Both FMS^wt^ and FMS^mut^ significantly increased the protein expression of TNFα and IL6 upon LPS stimulation (Fig. 3d). However, the levels of TNFα and IL6 were comparable between FMS^wt^- and FMS^mut^-transduced cells (Fig. 3d). These results suggest that FICD fragments may have minimal effects on inflammation, while FMS^mut^ is functionally active and can promote inflammatory responses. Next, we examined the role of c-FMS proteolysis in osteoclastogenic responses to the TNF family cytokine RANKL. M-CSF signaling is a key regulator of osteoclast differentiation^12^. As expected, ectopic expression of FMS^wt^ enhanced osteoclast differentiation and bone resorption compared with those of control cells (Fig. 3e). Strikingly, ectopic expression of FMS^mut^ led to diminished osteoclast formation relative to that of FMS^wt^-expressing cells (Fig. 3e), indicating that FMS^mut^ could not efficiently promote osteoclastogenesis in the same manner as FMS^wt^. Concomitantly, the increased bone resorption activity of FMS^wt^-expressing cells was also diminished in FMS^mut^-expressing cells (Fig. 3f). Therefore, our results suggested that increased FICD levels in FMS^wt^-expressing cells contribute to osteoclast differentiation and activity but have no effect on inflammatory responses.

### FICD knock-in mice exhibit an osteoporotic phonotype

**Fig. 4:**
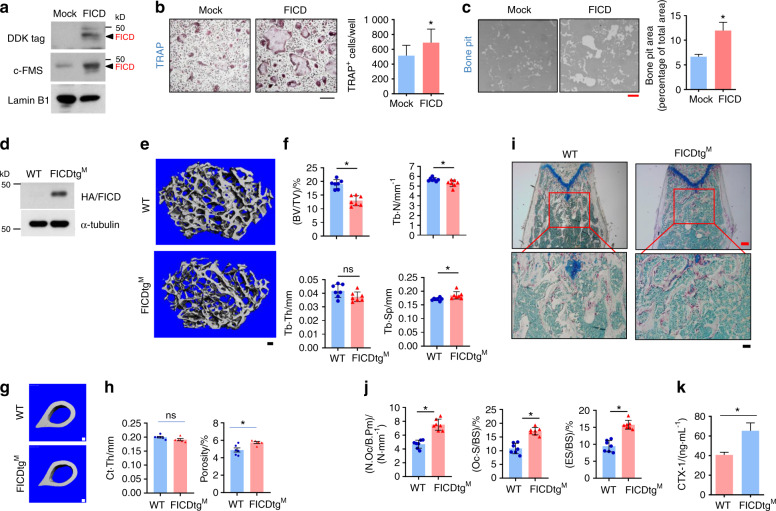
FICDtg^M^ mice exhibit an osteoporotic bone phenotype with increased osteoclast numbers. **a**, **c** BMDMs were transduced with retroviral particles encoding either control or FICD. **a** The protein expression of DDK-tagged FICD was determined by immunoblot analysis. **b** Osteoclastogenesis assay. The left panel shows representative images of TRAP-stained cells (*n* = 4). **c** Bone resorption activity analysis. The left panel shows representative images, and the right panel shows the percentage of the resorbed pit area per total area (*n* = 3). **d** BMDMs from wild type (WT) and FICDtg^M^ mice. Immunoblot analysis of the expression of HA-tagged FICD protein. **e–h** Micro-CT analysis of femurs from 12-week-old male wild-type (WT) and FICDtg^M^ mice (*n* = 7). Scale bar: 100 µm. **e** Representative images of distal femurs. **f** Bone parameters in distal femurs. Bone volume/tissue volume ratio (BV/TV), trabecular thickness (Tb.Th), trabecular number (Tb.N), and trabecular space (Tb.Sp) were determined by micro-CT analysis. **g** Representative images of cortical bone. **h** Cortical bone parameters. Cortical bone thickness (Ct.Th) and Cortical porosity. **i**, **j** Histomorphometric analysis of the distal femurs of 12-week-old male WT and FICDtg^M^ mice (*n* = 6). **i** Representative image showing TRAP-positive, multinucleated osteoclasts (red). **j** The number of osteoclasts per bone surface (N.Oc/BS), osteoclast surface area per bone surface (Oc.S/BS), and eroded surface per bone surface (ES/BS). **k** CTX-1 (WT = 5, FICDtg^M^ = 8) in the sera of WT and FICDtg^M^ mice. All data are shown as the mean ± SEM. ns, not significant. *P < 0.05 by two-tailed, unpaired *t*-test (**b**, **c**, **f**, **h**, **i**).

To further delineate the role of the FICD in osteoclasts, a DDK-tagged FICD was generated based on N-terminal sequencing and predicted protease cleavage sites by the GPS-CCD program^29^ (Supplementary Fig. 6). BMDMs were transduced with retroviral particles encoding DDK-tagged FICD. FICD protein expression was increased in FICD-transduced cells (Fig. 4a). Ectopic FICD expression enhanced RANKL-induced osteoclast differentiation and resorption compared with those of control cells (Fig. 4b, c), suggesting that constitutive FICD expression promotes osteoclastogenesis.

Increased FICD expression was observed in macrophages from RA patients (Fig. 1). To model the high expression of the FICD in vivo, we generated myeloid cell-specific conditional FICD knock-in mice (FICD^*KI/KI*^ x *Lyz2-cre*^*het*^ mice) by crossing FICD^*KI/KI*^ mice with myeloid cell-specific LysM-driven CRE recombinase mice (FICDtg^M^; Supplementary Fig. 7). FICD expression was measured by immunoblotting using anti-HA antibodies and was effectively increased in BMDMs (Fig. 4d). We examined whether the FICD regulates in vivo osteoclastogenesis. Micro-CT analysis showed that both FICDtg^M^ male and female mice exhibited decreased bone mass, and the bone volume/tissue volume (BV/TV) ratio and trabecular number (Tb.N) were significantly decreased compared with those of control mice (Fig. 4e, f, Supplementary Fig. 8a, b). Cortical thickness was comparable between control and FICDtg^M^ mice, while cortical porosity was higher in FICDtg^M^ mice (Fig. 4g, h). Histomorphometric analysis also showed that the number of osteoclasts, osteoclast surface area, and eroded surfaces were significantly higher in FICDtg^M^ mice than in control LysM cre (WT) mice (Fig. 4i, j). Accordingly, serum CTX was higher in FICDtg^M^ mice than in control mice (Fig. 4k). However, there were no differences in the mineral apposition rate (MAR) or bone formation rate (BFR) (Supplementary Fig. 8c–e). Overt phenotypic features, including body weight, spleen weight, and femur length, were not different between control and FICDtg^M^ mice (Supplementary Fig. 8f-i), suggesting that FICD overexpression in myeloid cells did not affect the gross phenotype. In addition, we generated FMScKOFICDtg^M^ mice by crossing c-FMS^f/fΔMx1^ mice with FICDtg^M^ mice. FICDtg^M^ mice on a c-FMS-null background also exhibited diminished bone mass compared with control c-FMS-null mice (Supplementary Fig. 9a, b) and showed increased in vivo osteoclast activity (Supplementary Fig. 9c, d). Overall, our findings suggest that FICD expression in macrophages results in decreased bone mass by increasing osteoclast numbers under physiological conditions.

### The FICD accelerates arthritis-induced bone erosion

**Fig. 5:**
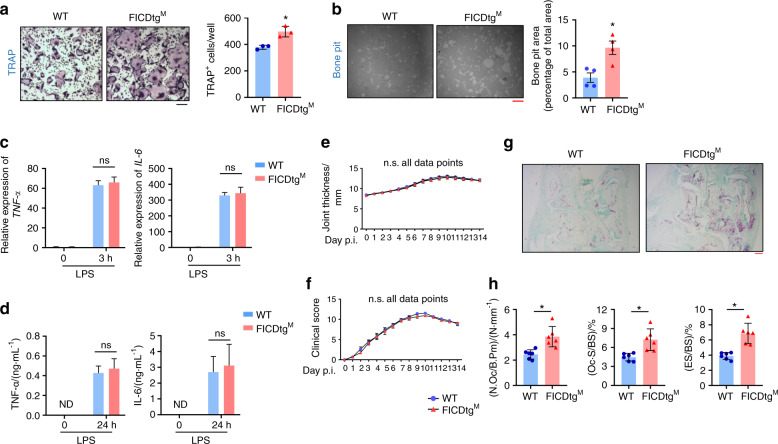
FICDtg^M^ mice show enhanced arthritic bone erosion. **a**, **b** BMDMs from WT and FICDtg^M^ mice were cultured with M-CSF and RANKL for 3 days. **a** Osteoclastogenesis assay. The left panel shows representative images of TRAP-stained cells. The right panel shows the percentage of TRAP-positive multinuclear cells per WT cell (*n* = 3). **b** Resorption pit assay. The left panel shows representative images, and the right panel shows the percentage of the resorbed pit area per total area (*n* = 3). BMDMs from WT and FICDtg^M^ mice were stimulated with LPS (10 ng·mL^−1^) for 3 h (**c**) and 24 h (**d**). **c** mRNA expression of *TNF-α* and *IL-6* was measured by q-PCR. **d** TNFα and IL6 protein levels in the culture media were measured by Luminex multiplex cytokine assays. **e**–**h** K/BxN serum transfer-induced arthritis model. Eight-week-old female wild-type and FICDtg^M^ mice were administered K/BxN serum on days 0 and 2. **e**, **f** Time course of joint swelling and clinical score analysis of serum-induced arthritis in littermate control and FICDtg^M^ mice (*n* = 6). (**g**) Representative images of the TRAP-stained tarsal bones (hind paws) of arthritic mice. **h** Histomorphometric analysis of tarsal bones. N.Oc/B.Pm Osteoclast number/bone parameter, Oc.S/BS osteoclast surface/bone surface, ES/BS eroded surface/bone surface. The black scale bar is 100 μm, and the red scale bar is 200 μm. All data are shown as the mean ± SEM. n.s. not significant. ND not detected. *P < 0.05 two-way ANOVA with *a post hoc Tukey* test (**c**–**f**) or two-tailed, unpaired *t*-test (**a**, **b**, **h**).

Given the high FICD levels in synovial CD14^+^ cells and its positive regulation of osteoclastogenesis without any effect on inflammatory responses, we hypothesized that the FICD may play a role in arthritic bone erosion. We first determined the effects of the FICD on inflammation and osteoclast differentiation in vitro. BMDMs from FICDtg^M^ mice or WT mice were cultured with M-CSF and RANKL to form osteoclasts in vitro. Consistent with the in vivo data, FICDtg^M^ cells showed significantly enhanced osteoclast differentiation and bone resorption activity relative to control cells (Fig. 5a, b). To examine the role of the FICD in inflammation, macrophages were stimulated with LPS (10 ng·mL^−1^). The LPS-induced mRNA and protein expression of TNFα and IL6 were comparable between FICDtg^M^ cells and control cells (Fig. 5c, d).

To address the importance of the FICD in osteoclast-mediated pathological bone resorption, we examined the effects of the FICD on bone erosion in a murine K/BxN serum-transfer-induced arthritis model^30^. K/BxN serum was administered intraperitoneally on days 0 and 2, and arthritis severity was assessed by clinical scores and ankle joint thickness until day 14. FICDtg^M^ mice exhibited minimal differences in joint swelling or inflammation compared with littermate control mice in K/BxN serum-induced arthritis (Fig. 5e, f). However, histomorphometric analysis revealed that osteoclast numbers, osteoclast surface areas, and the eroded surface in periarticular bone of FICDtg^M^ mice were significantly increased compared to those of WT mice (Fig. 5g, h). Thus, our results suggest that the FICD promotes pathological bone loss under inflammatory conditions in vivo.

### The FICD regulates RANKL-induced NFATc1 expression via the MNK1/2/eIF4E axis

**Fig. 6:**
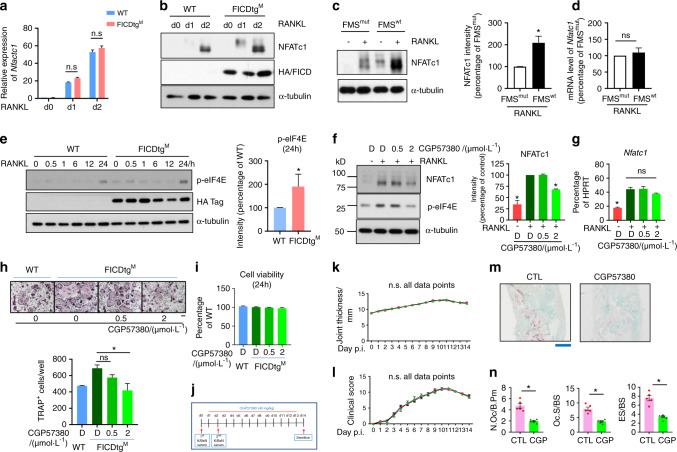
The FICD augments NFATc1 expression by activating the MNK1/2/eIF4E axis. **a**, **b** BMDMs from WT and FICDtg^M^ mice were stimulated with RANKL (50 ng·mL^−^1) for the indicated times. **a** RT-qPCR analysis of *Nfatc1* mRNA normalized to *Hprt* mRNA. **b** Immunoblot analysis with anti NFATc1, HA, or α-tubulin antibodies. **c**, **d** BMDMs from c-FMS^f/+^ Mx1-Cre mice were transduced with lentiviral particles encoding FMS^wt^ or FMS^mut^ and then cultured with M-CSF and RANKL. **c** Immunoblot analysis of whole cell lysate with anti-NFATc1 antibody. α-Tubulin was used as a control. The left panel shows representative images. The right panel shows the cumulative intensity of NFATc1 bands. The intensity of NFATc1 in FMS^mut^ was set as 100%. **d** The mRNA expression level of NFATc1. **e** Immunoblot analysis of whole cell lysates with phospho-eIF4E antibodies. HA-tagged FICD was detected by HA-antibodies. α-Tubulin was used as a control. The left panel shows representative images. The right panel shows the cumulative percentage of the intensity of the band (at 24 h) relative to the control from three independent experiments. **f**, **g** BMDMs from WT mice were treated with CPG57380 at the indicated doses and then cultured with RANKL for 1 day. D: DMSO. **f** Immunoblot analysis with anti-NFATc1, phospho-eIF4E, or α-tubulin antibodies. The left panel shows representative images. The right panel shows the cumulative percentage of the intensity of the band relative to the control (RANKL + DMSO) (*n* = 4). **g** *Nfatc1* mRNA expression was measured by qPCR relative to *Hprt* mRNA. the DMSO-treated RANKL condition was set as 100%. **h** Osteoclastogenesis assay. BMDMs from WT and FICDtg^M^ mice were treated with CPG57380 at the indicated doses and then cultured with RANKL for an additional 3 days. The upper panel shows representative images of TRAP-stained cells. The bottom panel shows the percentages of TRAP-positive multinuclear cells (MNCs: more than three nuclei) per control from three independent experiments. Scale bar: 100 μm. **i** Cell viability assay. BMDMs from WT and FICDtg^M^ mice were stimulated with CPG57380 at the indicated doses for 1 day. **j**–**n** K/BxN serum transfer-induced arthritis model. Nine-week-old male C57BL/6 J mice received K/BxN serum on days 0 and 2. Vehicle or CPG57380 (40 mg·kg^−1^, CPG) was administered intraperitoneally (i.p.) from day 2 until day 13. **j** Schematic diagram showing the experimental design. **k** Ankle thickness. **l** Arthritis score. **m** Representative images of TRAP-stained histological sections from the calcaneocuboid and tarsometatarsal joints. Scale bar: 1 mm. **n** Histomorphometric analysis of tarsal bones. N.OC/B.Pm Osteoclast number/bone parameter. OC.S/BS osteoclast surface/bone surface. ES/BS Eroded surface/bone surface. All data are shown as the mean ± SEM. CTL, Control. *P < 0.05; n.s. not significant by one-way ANOVA with *a post hoc Tukey* test (**a**, **f**–**i**, **k**, **l**) or two-tailed, unpaired *t*-test (**c**, **d**, **e**, **n**). The data represent at least three experiments.

To gain insight into the mechanism by which FICD fragments regulate osteoclastogenesis, we examined the effect of the FICD on the expression of NFATc1, a master regulator of osteoclastogenesis^31^. *Nfatc1* mRNA expression was comparable between WT and FICDtg^M^ mice (Fig. 6a). However, RANKL-induced NFATc1 protein levels were substantially increased in FICDtg^M^ cells compared with WT cells (Fig. 6b). Consistently, NFATc1 protein expression was diminished by impaired FICD generation in FMS^mut^ cells compared with FMS^wt^ cells, while *Nfatc1* mRNA expression was comparable between FMS^mut^ cells and FMS^wt^ cells (Fig. 6c, d). To explain the considerable discrepancy in *Nfatc1* mRNA and protein levels between WT and FICDtg^M^ osteoclasts, we examined the effect of the FICD on the activation of the mammalian target of the rapamycin (mTOR) pathway and on the induction of the MAPK interacting kinase (MNK1/2)-dependent pathway among several key signaling pathways that regulate protein translation^32^. We measured phospho-eIF4E as a downstream readout for the activation of the mTORC1 and MNK1/2 pathways. Strikingly, eIF4E phosphorylation was activated by RANKL stimulation and was significantly increased in FICDtg^M^ cells compared with WT cells (Fig. 6e), suggesting that the FICD may enhance eIF4E-dependent protein synthesis. To further delineate the cause of the increased eIF4E phosphorylation, we measured phospho-S6K and phospho-4EBP1 to determine the activation of the mTORC1 pathway. RANKL-induced mTORC1 activation was comparable between FICDtg^M^ and control cells (Supplementary Fig. 10a, b). Consistent with the literature^33^, NFATc1 protein expression was comparable between control cells and RAPTOR-deficient cells, which are a model for low mTORC1 signaling (Supplementary Fig. 10c). Our data suggest that the mTORC1 pathway does not regulate FICD-induced eIF4E phosphorylation. We next examined whether the MNK1/2-eIF4E axis regulates RANKL-induced NFATc1 expression using the MNK1/2 inhibitor CGP57380^34^. Inhibiting MNK1/2 activity indeed suppressed RANKL-induced NFATc1 protein expression in a dose-dependent manner in both mouse and human macrophages, whereas *Nfatc1* mRNA expression was marginally altered by CGP57380 treatment (Fig. 6f, g, Supplementary Fig. 10d, e). CGP57380 also suppressed osteoclast differentiation in a dose-dependent manner in CD14^+^ cells (Supplementary Fig. 10f). To examine the contribution of the MNK1/2 pathway to the increased osteoclastogenesis in FICDtg^M^ cells, we treated FICDtg^M^ cells with CGP57380. As expected, suppressing MNK1/2/p-eIF4E inhibited the enhanced osteoclastogenesis in FICDtg^M^ cells to levels comparable to those of WT osteoclasts (Fig. 6h). However, CGP57380 treatment induced minimal effects on cell viability (Fig. 6i). We examined the effect of CGP57380 on bone erosion in K/BxN serum transfer-induced arthritis. K/BxN serum was administered intraperitoneally on days 0 and 2, and then CGP57380 was administered after disease onset (Fig. 6j). Although the severity of arthritis was assessed by the clinical score and ankle joint thickness, and these factors were not affected by CGP57380 treatment (Fig. 6k, l), treatment with an MNK1/2 inhibitor decreased the number of osteoclasts and attenuated bone erosion in the K/BXN serum-induced arthritis model (Fig. 6m, n). Overall, our results suggest that increased phospho-eIF4E is a key regulator of increased osteoclastogenesis in FICDtg^M^ cells and that targeting the FICD/MNK1/2 axis significantly diminishes arthritic bone erosion.

### FICD/DAP5/Fxr1 complexes activate the MNK1/2 pathway and NFATc1 expression

**Fig. 7:**
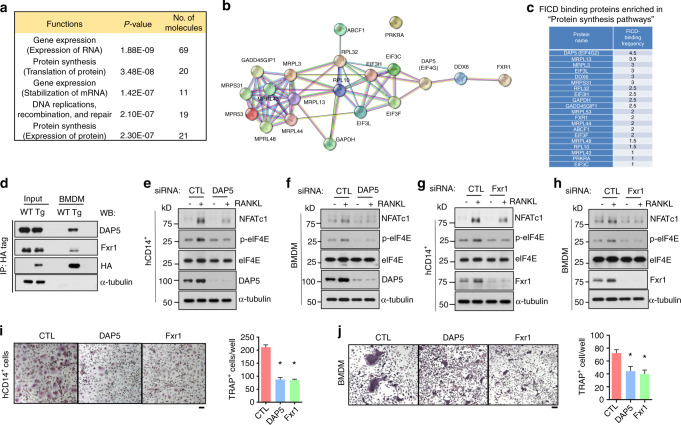
The FICD enhances the activation of MNK1/2/eIF4E via DAP5/Fxr1. **a** Ingenuity pathway analysis of 145 FICD-interacting proteins. Pooled data from two biological replicates were analyzed. **b** Interaction map showing 20 FICD-interacting proteins in “Protein synthesis pathways” using STRING functional protein association analysis. **c** Frequencies of the proteins shown in (**b**). **d** The interaction of the FICD with DAP5 or Fxr1 was determined by immunoblot analysis with anti-DAP5, Fxr1, HA, or α-tubulin antibodies. Whole cell lysates of BMDMs from WT and FICDtg^M^ mice were used for immunoprecipitation with anti-HA-tagged antibodies. Knockdown (KD) of DAP 5 (**e**, **f**, **i**, **j**) or Fxr1 (**g**, **h**, **i**, **j**) in both human CD14^+^ cells (**e**, **g**, **i**) and BMDMs (**f**, **h**, **j**). **e–h** The protein expression of NFATc1, p-eIF4E, eIF4E, DAP5, Fxr1, and α-tubulin was determined by immunoblot analysis. Osteoclastogenesis assay. KD of DAP5 or Fxr1 in hCD14^+^ cells (**i**) or in BMDMs (**j**) that were cultured with M-CSF and RANKL for 3 days. The left panel shows representative images of TRAP-stained cells. The right panel shows the percentages of TRAP-positive multinuclear cells (MNCs: more than three nuclei) per control from three independent experiments. CTL Control siRNAs. All data are shown as the mean ± SEM. *P < 0.05 by one-way ANOVA with *a post hoc Tukey test* (**i**, **j**). The data represent 2 biological replicates for mass spectrometry (**a**–**c**) and three independent experiments (**d**–**i**).

Next, we sought to identify the underlying mechanisms by which FICD increases eIF4E phosphorylation. We performed an unbiased proteomic analysis using mass spectrometry with two biological replicates to screen proteins that both interact with the FICD and regulate the MNK1/2 pathway. FICD-DDK was transfected into 293 T cells, and FICD-interacting proteins were immunoprecipitated using anti-c-FMS antibodies. A total of 145 FICD-interacting proteins were identified (Supplemental Table 1). Ingenuity pathway analysis showed that 20 FICD-interacting proteins were enriched in protein synthesis and posttranscriptional modifications (Fig. 7a). Among them, we focused on DAP5, which binds to MNK1 and is a part of protein translation initiation complexes^35^ (Fig. 7b, c). To verify the interaction between the FICD and DAP5, we performed immunoprecipitation analysis using BMDMs from wild-type and FICDtg^M^ mice. We found that the FICD bound to DAP5 (Fig. 7d). As Fxr1 was shown to form a complex with DAP5^36^, we also examined whether Fxr1 interacted with the FICD. The FICD also bound to Fxr1 (Fig. 7d), suggesting that the FICD might interact with the DAP5/Fxr1 complex.

As the function of the DAP5/Fxr1 complex in osteoclasts has not been previously characterized, we examined the effect of the DAP5/Fxr1 complex on osteoclastogenesis by knocking down these proteins in both human and murine osteoclasts using siRNAs. Both DAP5 and Fxr1 were increased upon RANKL stimulation, and knockdown of DAP5 and Fxr1 suppressed their expression in both human and murine macrophages (Supplementary Fig. 10g–j). Strikingly, DAP5 or Fxr1 deficiency suppressed RANKL-induced eIF4E phosphorylation and NFATc1 protein expression (Fig. 7e–h), suggesting that the FICD/DAP5/Fxr1 axis plays an important role in eIF4E phosphorylation and NFATc1 expression in osteoclasts. Accordingly, osteoclast differentiation was also suppressed by DAP5 or Fxr1 deficiency (Fig. 7i, j). Our data suggest that the DAP5/Fxr1 complex contributes to the activation of MNK1/2/eIF4E and NFATc1 expression in osteoclasts and serves as a positive regulator of osteoclast differentiation. Taken together, our findings suggest that the FICD promotes osteoclast differentiation by permitting the sustained activation of MNK1/2 and eIF4E phosphorylation, and in turn, NFATc1 expression is increased and enhances FICDtg^M^ osteoclast formation (Supplementary Fig. 11).

## Discussion

Cell surface receptors sense environmental stimuli and control cellular responses by activating downstream signaling cascades. However, recent studies have revealed that intramembrane cleavage of cell surface receptors also plays an important role in signaling processes and regulates cellular functions. Here, we demonstrated that c-FMS proteolysis was critically involved in the osteoclastogenic responses of macrophages to RANKL and cooperated with the conventional M-CSF/c-FMS signaling pathways. c-FMS is processed into smaller intracellular fragments (FICD fragments) in macrophages by engaging c-FMS-mediated signaling pathways. FICD fragments form a complex with DAP5 and activate the MNK1/2-eIF4E axis to enhance NFATc1 protein expression and osteoclastogenesis. Our data established the FICD as a positive regulator of osteoclastogenesis. Furthermore, by modeling the increase in FICD fragments in RA macrophages, myeloid cell-specific FICD expression enhanced in vivo osteoclastogenesis and promoted arthritic bone erosion in a murine arthritis model. These findings identify a novel function of c-FMS proteolysis in regulating (patho)physiological bone erosion and sensitivity to stimulation with the cytokine RANKL.

The altered expression of M-CSF and c-FMS has been implicated in the exacerbation of various diseases^37^. To readjust the c-FMS-M-CSF/IL-34 axis, several drug discovery programs have been focused on finding inhibitors of the tyrosine kinase activity of c-FMS^38^. Although inhibiting c-FMS kinase activity appears to be an attractive strategy and has already shown promise, the prolonged use of c-FMS inhibitors is limited by their side effects. Targeting osteoclasts using denosumab, an anti-RANKL antibody, shows efficacy on the progression of arthritic bone erosion without affecting RA disease activity^39,40^, emphasizing the importance of osteoclasts in arthritic bone erosion. A better understanding of osteoclast regulation in arthritis is important for developing osteoclast-specific therapeutic interventions for arthritic bone erosion. We demonstrated that FICD overexpression using transgenic FICD knock-in mice affected osteoclasts with no effect on disease activity, while inhibiting c-FMS signaling attenuated both disease activity and arthritic bone erosion in murine arthritis models. This finding was consistent with our observations that blocking c-FMS proteolysis had no effect on inflammation. In normal macrophages, the FICD level was very low. However, high FICD expression was found in RA synovial CD14^+^ cells, which have increased potential to differentiate into osteoclasts. Many plausible causes of arthritic bone erosion, including chronic inflammation, have been identified. In addition, predicative markers for arthritic bone erosion have been investigated. The correlation between serological markers and the radiographic progression in RA has been identified in a long-term longitudinal study, while some studies have shown a negative correlation between serological markers and bone destruction in RA^41–44^. We did not find a correlation between the levels of FICD fragments and serological markers in RA. Since our study had a small number of cohorts, further studies will be needed to determine whether the level of FICD fragments can be used as a predicative marker for arthritic bone erosion. Taken together, our findings revealed the pathophysiological importance of the FICD and its associated pathways in arthritic bone erosion and suggests that inhibiting FICD generation or function in RA patients who have high FICD expression in macrophages might be beneficial for inflammatory bone destruction.

Our results demonstrated that c-FMS proteolysis was involved not only in protein turnover but also in generating the necessary functional elements to promote osteoclastogenesis. High levels of M-CSF in the RA synovium and RA synovial fluids may contribute to c-FMS proteolysis and FICD generation. It has been shown that c-FMS proteolysis generates small fragments via TACE and ɣ-secreatase^10^. c-FMS proteolysis has been considered a disposal mechanism that is coupled with proteosomal degradation of c-FMS. Enhancing c-FMS degradation suppresses osteoclastogenesis^45–47^. When macrophages are exposed to inflammatory mediators, c-FMS is rapidly degraded, and osteoclastogenesis is suppressed by depleting RANK^45^. Inhibiting proteosomal degradation with bortezomib promotes c-FMS degradation and suppresses osteoclastogenesis^46,47^. However, the role of small fragments in macrophages/osteoclasts remains unknown. We identified the function of FICD fragments in myeloid cells under physiological and pathological conditions. Moreover, our data showed that the accumulation of FICD fragments was associated with pathological conditions such as RA. We also showed that FMS^mut^ did not generate FICD fragments and exhibited impaired osteoclastogenesis. Although we demonstrated that FMS^mut^ was a functional receptor, we could not exclude the potential effects of FMS^mut^ on other signaling pathways that play an important role in osteoclastogenesis. Thus, we used the FICD overexpression system in myeloid cells. Intriguingly, the effect of FICD fragments on the inflammatory response was negligible, suggesting the specific activity of the FICD on osteoclastogenesis. Our data from FICDtg^M^ mice suggest that impaired FICD generation in FMS^mut^ is likely to affect osteoclastogenesis. Although we screened the candidate protease that cleaves c-FMS using a protease inhibitor library that includes 53 known protease inhibitors, it is still possible that other proteases are involved in c-FMS proteolysis. Our study extended the current paradigm of the c-FMS signaling network by demonstrating that c-FMS proteolysis is a new player in the c-FMS signaling network.

The MNK1/2/p-eIF4E axis is a downstream mediator of FICD fragments and interacts with DAP5/Fxr1 complexes. The role of DAP5 and Fxr1 in osteoclasts has not been explored. We showed that DAP5 or Fxr1 deficiency suppressed NFATc1 expression and osteoclastogenesis. However, FICD/DAP5/Fxr1 complexes can target other proteins than NFATc1 to suppress osteoclastogenesis. Further investigation of the mechanisms by which c-FMS proteolysis regulates the function of DAP5 is needed for a deeper understanding. Moreover, inhibiting MNK1/2 activity suppressed osteoclastogenesis and bone erosion in a K/BXN serum-transfer arthritis model. Thus, our study provides important insights into the amenability of the FICD/DAP5/Fxr1/MNK1/2 axis to therapeutic intervention. In addition, blocking the pathways involved in the generation of the FICD can be a potential therapeutic strategy for osteoclast-mediated diseases. Overall, the effects of the FICD on osteoclast differentiation and bone resorption under pathological conditions can be determined by the levels of M-CSF, the activation of proteases, and the availability of FICD-interacting proteins.

In summary, our findings highlight the importance of c-FMS proteolysis in c-FMS-mediated signaling pathways in macrophages/osteoclasts and identify the mechanisms by which FICD generation and nuclear translocation occur. Our study also identifies a new pathway by which osteoclast differentiation and activity are enhanced in the pathogenesis of osteoclast-mediated bone diseases, which may be useful for developing innovative therapeutic interventions that specifically target osteoclast activity.

## Methods

Methods are available in the Supplementary material.

## Supplementary information

Supplemental information
