# Supplementary material for: Augmenting MNK1/2 activation by c-FMS proteolysis promotes osteoclastogenesis and arthritic bone erosion
Source: Bone Res. 2021 Oct 20;9:45. doi: 10.1038/s41413-021-00162-0 (PMC8528869; doi:10.1038/s41413-021-00162-0)
Supplement: Supplementary file 1 — Supplemental information [file 41413_2021_162_MOESM1_ESM.pdf]

Supplemental Information

Augmenting MNK1/2 Activation by c-FMS Proteolysis Promotes Osteoclastogenesis and Arthritic Bone Erosion

Se Hwan Mun, Seyeon Bae, Steven Zeng, Brian Oh, Carmen Chai, Matthew Jundong Kim, Haemin Kim, George Kalliolias, Chitra Lekha Dahia, Younseo Oh, Tae-Hwan Kim, Jong Dae Ji, and Kyung-Hyun Park-Min*

***Corresponding author. Email: ParkminK@hss.edu; 212-774-7631**

**Supplemental methods**	**2 - 7**
**Supplemental References**	**8**
**Supplemental Figures 1-10**	**9 - 22**
**Supplemental Table 1**	**23**
**Supplemental Table 2**	**24**

Material and Methods

Mice

Human c-FMS fragment (FICD) knock-in mice ($FICD^{KI/KI}$) were generated and purchased from Cyagen Biosciences Inc. (Guangzhou, Guanddong, China). Briefly, mouse genomic fragments containing homology arms of ROSA26 allele were amplified from BAC clone using PCR and Neo (positive selection marker) flaked by SDA (self-deletion anchor) and CAG-loxP-3*polyA-loxP, and human CSF1R intracellular domain-poly A cassette (NM_005211.3) were assembled into targeting vector shown in Supplemental Figure 5A. Targeting vector (pRP.ExBiEF1A-loxp-stop-loxp-hFICD) were then linearized by Not I digestion and electrophorated into C57BL/6J ES cells. Six positive G418 resistant ES clones were selected and further confirmed by Southern blot (Supplemental Figure 5B). The G418-resistant ES clones were then transfected with FLP (flippase) to remove the Neo drug marker. Targeted ES cells were injected into mouse blastocysts and were transferred into surrogate mothers. Male chimera was bred with C57BL/6J female to generate F1 heterozygous mice. F1 mice were crossed each other to generate wild type, heterozygous, and homozygous ($FICD^{KI/KI}$) mice. $FICD^{KI/KI}$ mice were crossed to Lys2-Cre mice (The Jackson Laboratory) to generate $FICD^{KI/KI}$ LysM-Cre ($LysM^{cre/+}$x $FICD^{KI/KI}$) mice. Age- and gender matched littermate $LysM^{cre/+}$ $FICD^{+/+}$mice were used as controls. 8 week-old female $LysM^{cre/+}$x $FICD^{KI/KI}$ mice were randomly assigned for K/BXN serum transfer model, while 12 week-old male $LysM^{cre/+}$x $FICD^{KI/KI}$ mice were used for micro-CT analysis. C57BL/6J female mice were obtained from Jackson Laboratory and were randomly allocated for in vitro experiments. Both $Csf1r^{fl/fl}$ mice and Mx1-Cre transgenic mice were purchased from The Jackson Laboratory. $Csf1r^{fl/fl}$ mice were crossed to Mx1-Cre transgenic mice to generate Mx1 Cre $Csf1r^{fl/+}$ mice to diminish intracellular FICD generation. c-$FMS^{flox/+}$ Mx1cre(+) mice (referred to as c-$FMS^{het\Delta MX}$ mice) and littermate control c-$FMS^{WT/WT}$ Mx1cre(+) mice were used for the experiments. To induce *FMS* deletion, 300 mg of Poly (I:C) (Thermo Fisher Scientific) was injected three times at age of 6 weeks. LysM Cre mice were crossed with $Raptor^{fl/fl}$ mice to generate either $LysM^{Cre/Cre}$ $Raptor^{+/+}$or $LysM^{Cre/Cre}$ $Raptor^{+/+}$ mice. All animals were randomly assigned into experimental groups. Animals were housed in a specific pathogen-free environment in the Weill Cornell Medicine vivarium and all the experiments conformed to the ethical principles and guidelines approved by the Institutional and Animal Care and Use Committee of Weill Cornell Medical College.

Human studies

Human synovial fluid (SF) samples were collected from RA and osteoarthritis (OA) patients as previously described[1]. Patients SF from active effusions was obtained from 24 patients with RA, and 10 patients with OA. The protocol was approved by the Hospital for Special Surgery Institutional Review Board (2016-957, 2016-958, and 2016-139). Active effusion was defined as an acute noninfectious inflammatory SF accumulation attributed to a flare of RA that required arthrocentesis based on medical indications. The diagnosis of RA was based on the 1987 revised criteria of the American College of Rheumatology[2]. There was limited information about patients' medications, and correlation of our findings with therapy was not possible.

The experiments for Supplementary Figure 2 were approved by the Ethics Committee of Hanyang University Hospital for Rheumatic Disease (IRB No. 2008-09-001 and IRB No. 2017-05-003). All RA patients provided their written informed consent to participate in this study. The patients with RA were tested for erythrocyte sedimentation rate (ESR) with the Westergren method and for serum C-reactive protein (CRP) with an immunoturbidimetric method (mg/dl). Anti-cyclic citrullinated peptide (anti-CCP) antibodies were measured with the ImmuLisa CCP enzyme-linked immunosorbent assay (U/ml) (IMMCO Diagnostics, Buffalo, NY). Spearman's rank correlation coefficient test was employed to evaluate the correlation of assessed parameters. The level of significance was set at $P < 0.05$.

Cells
Peripheral blood mononuclear cells (PBMCs) from blood leukocyte preparations purchased from the New York Blood Center or mononuclear cells from SF of RA patients were isolated by density gradient centrifugation with Ficoll (Invitrogen, Carlsbad, CA). $CD14^+$ cells were obtained by isolation using anti-CD14 magnetic beads, as recommended by the manufacturer (Miltenyi Biotec, CA). Human $CD14^+$ cells were cultured in α-MEM medium (Invitrogen) supplemented with 10 % fetal bovine serum (FBS, Hyclone; SH30070.03) and 1% L-glutamin with 20 ng/ml of M-CSF for 12 hours to generate osteoclast precursor cells (OCPs) The purity of monocytes was >97%, as verified by flow cytometric analysis [3].

For human osteoclastogenesis assays, cells were added to 96 well plates in triplicate at a seeding density of $5x10^4$ cells per well. Osteoclast precursors were incubated with 20 ng/ml of M-CSF and 40 ng/ml of human soluble RANKL up to 5 days in α-MEM supplemented with 10 % FBS and 1% L-glutamine. Cytokines were replenished every 3 days. On each day, cells were fixed and stained for TRAP using the Acid Phosphatase Leukocyte diagnostic kit (Sigma; 387A) as recommended by the manufacturer. Multinucleated (greater than 3 nuclei), TRAP-positive osteoclasts were counted in triplicate wells. For mouse osteoclastogenesis, bone marrow (BM) cells were flushed from femurs of mice and cultured with murine M-CSF (20 ng/ml) on petri dishes in α-MEM supplemented with 10% FBS, 1% anti-biotics and 1% L-glutamin after lysis of RBCs using ACK lysis buffer (Gibco). Then, the non-adherent cell population was recovered the next day and cultured with M-CSF-containing conditional medium (CM) for three additional days. We defined this cell population as mouse BMDMs. Then, we plated $2x10^4$ cells per well in triplicate wells on a 96 well plate and added M-CSF (20 ng/ml) and RANKL (50 ng/ml) up to 4 days, with exchange of fresh media every 3 days. All cell-cultures were performed by a modification of previously published method [3].

RNA preparation and real-time PCR
DNA-free RNA was obtained using the RNeasy Mini Kit from QIAGEN with DNase treatment, and 0.5 μg of total RNA was reverse transcribed using a First Strand cDNA Synthesis kit (Fermentas, Hanover, MD). Real time PCR was performed in triplicate using the iCycler iQ thermal cycler and detection system (Applied Biosystems, Carlsbad, CA) following the manufacturer's protocols. The primer sequences are listed in Supplemental Table 2.

Enzyme-linked immunosorbent assay (ELISA)
c-FMS and M-CSF in synovial fluids and the supernatants of cell culture was measured using sandwich ELISA kit (R&D Systems; DY329 and DY216) according to manufacturer's instructions. C-telopeptide of type I collagen (CTX-1) and Procollagen 1 N-Terminal Propeptide (P1NP) in Serum from WT and FICDtgM mice were measured using RatLapstm EIA kit (Immunodiagnostic Systems; AC-06F1) according to the manufacturer's instructions.

Measurement of cytokine production
IL-6 and TNF-α, in culture supernatants were assessed quantitatively by Luminex multiplex cytokine assay (R&D Systems) as described by the manufacturer.

RNA Interference
RNA interference 0.2 nmol of three short interfering RNAs (siRNAs), specifically targeting human TACE (Invitrogen; HSS186181), CAPAN 1(Dharmacon; L005799-00-0005), CAPAN 5(Dharmacon: L-009423-00-0005), CAPAN 6(Dharmacon: L-009423-00-0005) or control siRNA (D-001810-10) were transfected into primary human $CD14^+$ monocytes with the Amaxa Nucleofector device set to program Y-001 using the Human Monocyte Nucleofector kit (Amaxa), as previously described [4].

Bone marrow derived macrophages (BMDMs) from wild type mice were plated 2 x 10^5

/ml (24 well plate) and were cultured with 20ng/ml M-CSF for 24 hours. Cells were transfected with the 100nM siRNA mouse Control DAP5 (Dharmacon: L-064521-00-0005) or Fxr1(Dharmacon: L-045530-00-0005) with TransIT-TKO® Transfection Reagent /opti-men. After 4hours, add 500 ul Opti-MEM with M-CSF (20ng/ml) and FBS(final con. 5%) serum 48hours. and then changed complete medium with 10ng/ml M-CSF and 50ng/ml RANKL for 24hours. Cells were lysed.

Immunoblot

Whole cell extracts were prepared by lysis in buffer containing 1x Lamin sample buffer (Bio-rad) and 2-Mercaptoethanol (Sigma) or RIPA buffer (Sigma). The cell membrane-permeable protease inhibitor, Pefablock (1 mM), was added immediately prior to harvest cells. The membrane proteins were extracted with Mem-PER™ Plus Membrane Protein Extraction Kit (Thermorfisher scientific; 89842) according to manufacturer's instructions. To extracts of nucleus protein, cells were incubated in buffer A (10 mM Hepes, pH 7.9, 1.5 mM $MgCl_2$, 10 mM KCl, 0.1mM EDTA, 0.1mM EGTA, proteinase inhibitor cocktail (Complete, Roche) and 1mM DTT) for 15 min at 4°C. NP-40 was added to a final concentration of 0.5%. Nucleus was collected by centrifugation (5000 rpm, 5 min). The pellets were lysed by Bioruptor -ultrasonicator (UCD400, Diagenode) in buffer B (20 mM Hepes, pH 7.9, 0.4M NaCl, 10 mM KCl, 1mM EDTA, 1mM EGTA, 10% Glycerol, proteinase inhibitor cocktail and 1mM DTT) and collected the supernatant by centrifugation (12,000 rpm, 10 min). The protein concentration of nuclear extracts was quantitated using the Bradford assay (Bio-Rad; 5000001). For immunoblot, proteins were separated on 7.5 or 10% SDS-PAGE gels, transferred to polyvinylidene difluoride membranes (PVDF, Millipore; ISEQ00010), and detected by antibodies as listed in the figure legends.

Lentiviral and Retroviral transduction

The vector containing full-length mouse c-FMS (MR211364, FMS^{wt}) or TACE cleavage resistant c-FMS (FMS^{mut}) were purchased from Blue Heron Biotech, LLC (Origene, MD). Briefly, FMS^{mut} generated by switching 14 amino acids from TACE cleavage sites of c-FMS with sequences from insulin receptor extracellular regions [5]. The target sequences were shown in Figure 3C. FMS^{mut} and FMS^{wt} were cloned into pLenti-EF1a-C-Myc-DDK-IRES-Puro vector. 293T cells were transfected with pUC-MDG, pCMV8.9, and an empty vector [6], FMS^{wt}, or FMS^{mut} by Lipofectamin3000 regent (Invitrogen) to generate lentiviral particles. Supernatants were collected and concentrated with Lenti-X ™ Concentrator (TaKaRa Clontec.). BMDMs from $Csf1r^{f/+}$ Mx1-Cre^{+} male mice were cultured for 2 days with M-CSF, and then cells were transduced with lentiviral particles with 8 μg/mL polybrene (Santacruz; sc-134220). After 24h, infected cells were re-plated for osteoclastogenesis experiment. FICD gene was amplified using PCR primers from the human c-FMS cDNA with the following primers: FICD-C; forward: 5'-GGGTCTAGAATGTCCGAGCTGAAGATC-3' and reverse: 5'-GGGATACCGACTGCATTAAT GCTGTT-3'. For retrovirus transduction, FICD genes were ligated into the retroviral vector, pMX-puro (Cell Biolabs) to generate pMX-puro-FICD. The retroviral vector pMXs-puro-FICD and its control vector were transfected into a packaging cell line, Plat-E, using FuGENE HD Transfection Reagent (Promega), and then the viral supernatant was collected after 24 hours of incubation. The filtered virus-containing supernatant was mixed 6 μg/mL polybrene (Santacruz) along with 10% of M-CSF-containing conditional medium, and then added to cells. After 48 hours of viral incubation, cells were re-plated for experiments[7].

Flow Cytometry

Lentiviral vector encoding control, FMS^{wt} and FMS^{mut} were used for transducing 293T cells. Cells were stimulated by 12-Otetradecanoylphorbol-13-acetate (TPA, 100 ng/ml) for 30 mins and were stained with isotype-PE (mouse IgG2a) or anti c-FMS-PE antibodies. Stained each

cells were performed with a FACS Canto (BD Biosciences) and analyzed with FlowJo software (Tree Star Inc.).

Bone-resorption pit assays

Bone-resorption activity of osteoclasts was examined using 96-well Corning Osteo Assay Surface plates (Sigma). Mouse OCPs were plated at a seeding density of $1x10^4$ per well and incubated with M-CSF (20ng/ml) and RANKL (50 ng/ml) for 5 days, with exchange of fresh M-CSF and RANKL every two days. After removing cells with 10% bleach solution, plates were stained with 1% toluidine blue solution to visualize the formation of pits. Resorbed area was analyzed using OsteoMeasure software (OsteoMetrics, Inc.).

MASS spectrophotometer assay

293T cells were transfected with pCMV6-Entry-c-FMS-MYC-DDK (NM_005211, Origene, Rockville, MD) using Lipofectamin 3000 (Thermofisher scientific). Transfected cells were incubated with M-CSF (20ng/ml) for one day and nuclear proteins were immunoprecipitated (IP) with antibodies against N-terminal of c-FMS (Santa Cruz; H-300 and R&D systems; clone #61780) to remove full-length c-FMS as a negative selection. Subsequently, the IP-proteins were incubated with either mouse IgG or DDK-tag Ab conjugated magnetic bead (Origene). Proteins bound to ab-beads were eluted with water. Samples were subjected to SDS PAGE gel was submitted for the mass spectrophotometery assay. Mass Spectrometry assay (n=2) was performed by The Taplin Biological Mass Spectrometry Facility in Harvard Medical School. Briefly, excised gel bands were cut into approximately 1 mm^3 pieces. Gel pieces were then subjected to a modified in-gel trypsin digestion procedure[8]. Gel pieces were washed and dehydrated with acetonitrile for 10 min. followed by removal of acetonitrile. Pieces were then completely dried in a speed-vac. Rehydration of the gel pieces was with 50 mM ammonium bicarbonate solution containing 12.5 ng/µl modified sequencing-grade trypsin (Promega, Madison, WI) at 4ºC. After 45 min., the excess trypsin solution was removed and replaced with 50 mM ammonium bicarbonate solution to just cover the gel pieces. Samples were then placed in a 37ºC room overnight. Peptides were later extracted by removing the ammonium bicarbonate solution, followed by one wash with a solution containing 50% acetonitrile and 1% formic acid. The extracts were then dried in a speed-vac (~1 hr). The samples were reconstituted in 5 - 10 µl of HPLC solvent A (2.5% acetonitrile, 0.1% formic acid). A nano-scale reverse-phase HPLC capillary column was created by packing 2.6 µm C18 spherical silica beads into a fused silica capillary (100 µm inner diameter x ~30 cm length) with a flame-drawn tip[9]. After equilibrating the column each sample was loaded via a Famos auto sampler (LC Packings, San Francisco CA) onto the column. A gradient was formed and peptides were eluted with increasing concentrations of solvent B (97.5% acetonitrile, 0.1% formic acid). As peptides eluted they were subjected to electrospray ionization and then entered into an LTQ Orbitrap Velos Pro ion-trap mass spectrometer (Thermo Fisher Scientific, Waltham, MA). Peptides were detected, isolated, and fragmented to produce a tandem mass spectrum of specific fragment ions for each peptide. Peptide sequences (and hence protein identity) were determined by matching protein databases with the acquired fragmentation pattern by the software program, Sequest (Thermo Fisher Scientific, Waltham, MA)[10]. All databases include a reversed version of all the sequences and the data was filtered to between a one and two percent peptide false discovery rate.

The Ingenuity Pathway Analysis (IPA)
IPA was used to analyze the functions of FICD-interacting proteins obtained from mass spectrophotometry. The molecular and cellular function was used to predict the functions whose change in enrichment relative to control could explain the interaction with FICDs.

Immunocytochemistry
Human $CD14^+$-monocytes were cultured with M-CSF (20ng/ml) in culture slide (BD Falcon; REF 354104) for 2days. Cells were fixed with 3.7% formalin in PBS for 20 min at room temperature. Cells were permeabilized with 1% triton X-100 for 5min, washed 3 times before blocking with solution that contains 5% horse serum, 5% Goat and 1% BSA (without IgG) in PBS for 1h. Cells were incubated with primary antibody C-terminus specific c-FMS Ab (SantaCruz Biotechnology; sc-692) overnight at 4°C followed by incubation with anti-rabbit Alexa Fluor 488-conjugated secondary antibody (A11008, Thermo Fisher Scientific) for 40 min in room temperature. After washed, finally cells were mounted with ProLongTMGold antifade regent with-DAPI (P36931, Invitrogen). The stained cells were imaged using a Zeiss Axioplan microscope (Zeiss) with an attached Leica DC 200 digital camera (Leica) or a confocal microscope system (Zeiss LSM 880, Laser excitation/emission: 405/425 and 488/525). To determine c-FMS in the nucleus, confocal three-dimensional Z-stacks were acquired for each sample using a a Plan-Apochromat 63 × /1.4 oil Dic M27 objective (Zeiss, Germany) with a slice of increment of 0.5 µm. The images were processed with Image j-Fiji software.

Immunoprecipitation
2 x10^6 BMDMs from $LysM^{cre/cre}$ mice were seeded into 100mm dish and were incubated with M-CSF (10ng/ml) for overnight. Cells were treated with 50ng/ml RANKL for one additional day and lysed with RIPA buffer with proteinase inhibitor cocktail. An equal amount of cell lysates were incubated with magnetic beads conjugated with anti HA-Tag antibody (Thermo Fisher scientific; 88836) for 24h at 4 °C. The beads were washed 5 times with washing buffer (20 mM HEPES [pH 7.5], 150 mM NaCl, 0.1% NP-40, 1% glycerol, protease and phosphatase inhibitors). Proteins eluted from the bead with elution buffer (pH 2.8, Prod#1858606). The sample were incubated in 95 °C for 10 mins and then were analyzed by immunoblotting.

Micro-CT and histomorphometry analysis
µ-CT analysis[11] was performed as described previously[12], and all samples were included in the analysis conducted in a blinded manner. For µCT analysis, prior to decalcification, femurs with intact joints were scanned by microCT, with an isotropic voxel resolution of 6 µm (µCT35, Scanco, Bruttisellen, Switzerland; 55kVp, 145µA, 600ms integration time) to evaluate morphological changes in bone. Bone morphology in the femur was examined in two regions: the diaphysis and the metaphysis. For cortical bone, the volume of interest (VOI) encompassed cortical bone within a 231-slice section in the diaphysis. For trabecular bone, the VOI encompassed a 200-slice section in the metaphysis, proximal to the growth plate. To ensure exclusion of primary spongiosa in the growth plate, VOIs began 50 slices proximal to the median of the growth plate. Representative trabecular images were composed of the 100 slices either proximal or distal to the growth plate. Outcome parameters for cortical bone included thickness and tissue mineral density (TMD). Trabecular bone parameters included bone volume fraction (BV/TV), trabecular thickness (Tb.Th), trabecular separation (Tb.Sp), and trabecular TMD. 3D reconstructions were generated by stacking thresholded 2D images from the contoured region.

Histomorphometry experiment was performed with tarsal bone of vehicle or MDL28170 treated mice. Bone histomorphometric analysis was performed in a blinded, nonbiased manner using a computerized semi-automated system (Osteomeasure, TN) with light microscopy. The tarsal bones were fixed in 4% paraformaldehyde for 2 days, were decalcified with 10% neutral

buffered EDTA (Sigma-Aldrich), and were embedded in a paraffin. The quantification of osteoclast was performed in paraffin embedded tissues that were stained for TRAP and Methyl green (Vector Laboratories). Osteoclast cells were identified as multinucleated TRAP-positive cells adjacent to bone. The measurement terminology and units used for histomorphometric analysis were those recommended by the Nomenclature Committee of the American Society for Bone and Mineral Research[13].

Bone formation assay
To assess dynamic histomorphometry, mice received intraperitoneal injections of 10 mg/kg of calcein, 5 days and 2 days before death. Bones were fixed for one day in 4% paraformaldehyde. After decalcification for one day, bones were incubated in 30 % sucrose overnight and subsequently embedded in OCT compound (Thermo Fischer Scientific, Waltham, MA). Mineral apposition rate (MAR) and Bone formation rate (BFR) were analyzed by a computerized semi-automated system (Osteomeasure, TN) with fluorescent microscopy.

K/BXN serum transfer arthritis model
For arthritis experiments, K/BxN serum pools were prepared as described previously [14]. Arthritis in 8-week-old C57BL/6J male mice (The Jackson Laboratory) was induced by intraperitoneal injection of 100 µl of K/BxN serum on days 0 and 2. . To analyze the effect of MDL28170 and CGP57380, the mice were randomized and treated with either vehicle (n=10), MDL28170 (10 mg/kg) or CGP57380 (40mg/kg) with intraperitoneally (i.p) every day for 11 or 13 days. Vehicle or MDL28170 were prepared in 2.5% DMSO and 10% KLEPTOSE pH7.0 (Roquette Phama). CGP57380 was prepared in 4% DMSO and 30% PEG300 (Selleckchem) in 0.9% saline solution (BD science)[15]. The development of arthritis was monitored by measuring the thickness of wrist and ankle joints using dial-type calipers (Bel-Art Products) and scoring the wrist and ankle joints. For each animal, joint thickness was calculated as the sum of the measurements of both wrists and both ankles. Joint thickness was represented as the average for every treatment group. The severity of arthritis was scored in a blinded fashion by four investigators for each paw on a 3-point scale, in which 0 = normal appearance, 1 = localized edema or erythema over one surface of the paw, 2 = edema or erythema involving more than one surface of the paw, 3 = marked edema or erythema involving the whole paw. The scores of all four paws were added for a composite score [16].

QUANTIFICATION AND STATISTICAL ANALYSIS
Graphpad Prism 8.0 for Windows was used for all statistical analysis. Detailed information about statistical analysis, including tests and values used, and number of times experiments were repeated is provided in the figure legends. *P* values are provided in the text or the figure legends. Shapiro-Wilk normality tests were performed and for data that fell within Gaussian distribution, we performed appropriate parametric statistical tests and for those that did not fall within equal variance-Gaussian distribution, we performed appropriate non-parametric statistical tests. $P < 0.05$ (*) was taken as statistically significant. Sample sizes were chosen according to standard guidelines. Number of animals was indicated as ‘‘n.’’

Supplementary References

1 Gordon, R. A., Grigoriev, G., Lee, A., Kalliolias, G. D. & Ivashkiv, L. B. The interferon signature and STAT1 expression in rheumatoid arthritis synovial fluid macrophages are induced by tumor necrosis factor alpha and counter-regulated by the synovial fluid microenvironment. *Arthritis and rheumatism* **64**, 3119-3128, doi:10.1002/art.34544 (2012).

2 Arnett, F. C. *et al.* The American Rheumatism Association 1987 revised criteria for the classification of rheumatoid arthritis. *Arthritis and rheumatism* **31**, 315-324 (1988).

3 Park-Min, K. H. *et al.* Inhibition of osteoclastogenesis and inflammatory bone resorption by targeting BET proteins and epigenetic regulation. *Nature communications* **5**, 5418, doi:10.1038/ncomms6418 (2014).

4 Park-Min, K. H. *et al.* FcgammaRIII-dependent inhibition of interferon-gamma responses mediates suppressive effects of intravenous immune globulin. *Immunity* **26**, 67-78, doi:10.1016/j.immuni.2006.11.010 (2007).

5 Vahidi, A., Glenn, G. & van der Geer, P. Identification and mutagenesis of the TACE and gamma-secretase cleavage sites in the colony-stimulating factor 1 receptor. *Biochemical and biophysical research communications* **450**, 782-787, doi:10.1016/j.bbrc.2014.06.061 (2014).

6 Ohno, H. *et al.* A contact investigation of the transmission of Mycobacterium tuberculosis from a nurse working in a newborn nursery and maternity ward. *Journal of infection and chemotherapy : official journal of the Japan Society of Chemotherapy* **14**, 66-71, doi:10.1007/s10156-007-0565-0 (2008).

7 Bae, S. *et al.* MYC-dependent oxidative metabolism regulates osteoclastogenesis via nuclear receptor ERRalpha. *The Journal of clinical investigation* **127**, 2555-2568, doi:10.1172/JCI89935 (2017).

8 Shevchenko, A., Wilm, M., Vorm, O. & Mann, M. Mass spectrometric sequencing of proteins silver-stained polyacrylamide gels. *Anal Chem* **68**, 850-858 (1996).

9 Peng, J. & Gygi, S. P. Proteomics: the move to mixtures. *J Mass Spectrom* **36**, 1083-1091, doi:10.1002/jms.229 (2001).

10 Eng, J. K., McCormack, A. L. & Yates, J. R. An approach to correlate tandem mass spectral data of peptides with amino acid sequences in a protein database. *J Am Soc Mass Spectrom* **5**, 976-989, doi:10.1016/1044-0305(94)80016-2 (1994).

11 Bouxsein, M. L. *et al.* Guidelines for assessment of bone microstructure in rodents using micro-computed tomography. *J Bone Miner Res* **25**, 1468-1486, doi:10.1002/jbmr.141 (2010).

12 Shim, J. H. *et al.* Schnurri-3 regulates ERK downstream of WNT signaling in osteoblasts. *J Clin Invest* **123**, 4010-4022, doi:10.1172/JCI69443 (2013).

13 Parfitt, A., Drezner MK, Vlorieux FH, Kanis JA, Malluche H, Meunier PJ, Ott SM, Recker RR. Bone histomorphometry:stardization of nomenclature, symbols, and units. Report of the ASBMR Histomorphometry Nomenclature Committee. *J. Bone Mineral Research* **2**, 595-610 (1987).

14 Korganow, A. S., Weber, J. C. & Martin, T. [Animal models and autoimmune diseases]. *Rev Med Interne* **20**, 283-286, doi:10.1016/s0248-8663(99)83060-4 (1999).

15 Lim, S. *et al.* Targeting of the MNK-eIF4E axis in blast crisis chronic myeloid leukemia inhibits leukemia stem cell function. *Proceedings of the National Academy of Sciences of the United States of America* **110**, E2298-2307, doi:10.1073/pnas.1301838110 (2013).

16 Murata, K. *et al.* Hypoxia-Sensitive COMMD1 Integrates Signaling and Cellular Metabolism in Human Macrophages and Suppresses Osteoclastogenesis. *Immunity* **47**, 66-79 e65, doi:10.1016/j.immuni.2017.06.018 (2017).

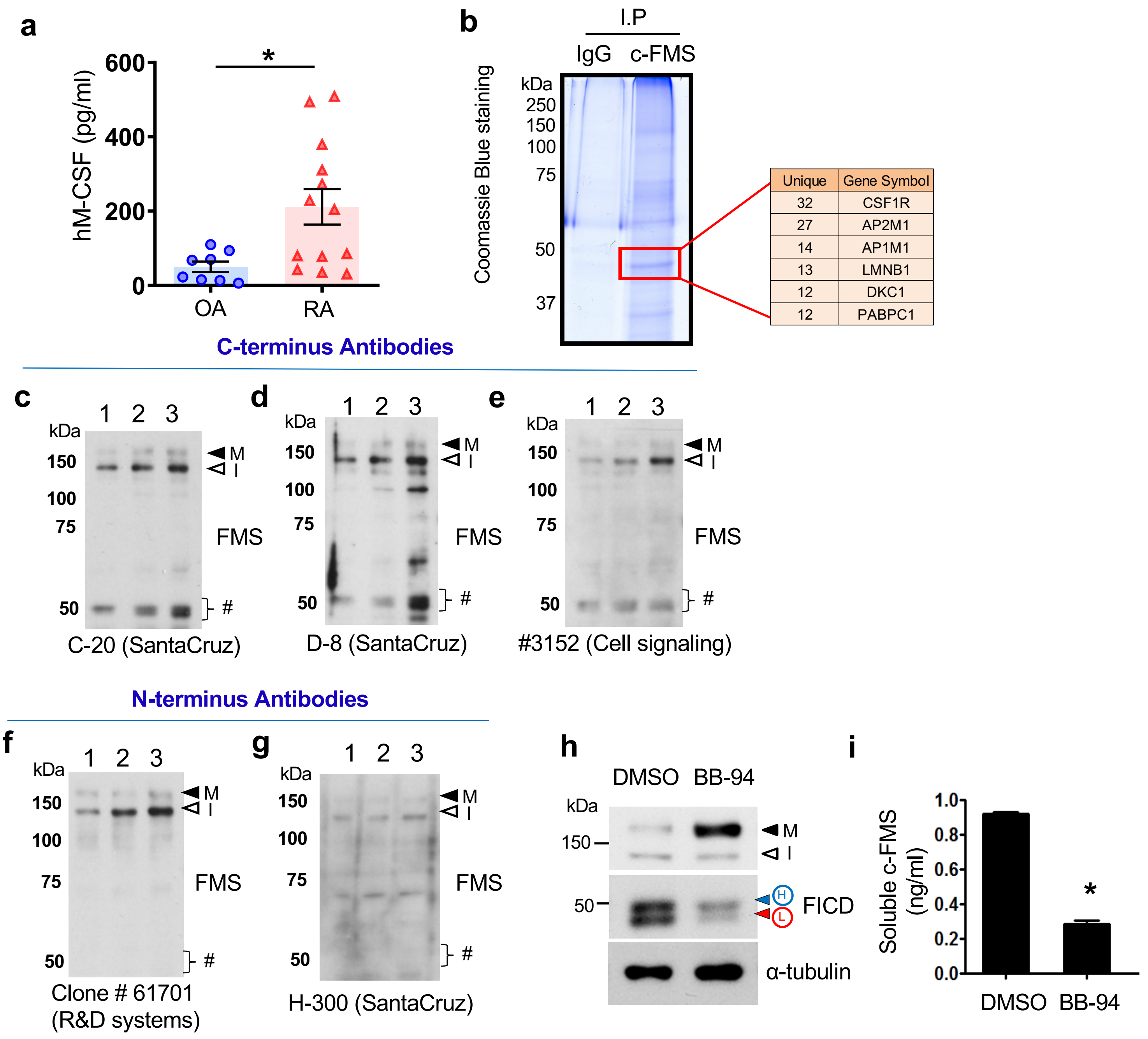

Supplementary Fig. 1. c-FMS expression in synovial macrophages.
(**a**) The levels of M-CSF in synovial fluids from patients with rheumatoid arthritis (RA, n=13) and osteoarthritis (OA, n=8) were measured. (**b**) Mass spectrometry analysis for proteins from ~50kDa bands (red box) identified c-FMS as one of top genes. An image of coomassie blue stained genes showing immunoprecipitated cells lysates with either anti-DDK-tag antibodies or IgG control. (**c**-**g**) Human CD14+ cells were cultured with M-CSF (20ng/ml). Immunoblot of c-FMS with different antibodies against C-terminal of c-FMS; (**c**) C-20, (**d**) D-8, and (**e**) #3152. Immunoblot of c-FMS with different antibodies against N-terminal of c-FMS; (**f**) # 61701 , (**g**) H-300. (**h**-**i**) Human CD14+ cells were cultured with M-CSF (20 ng/ml) for 8 hours to induce early signals, and then BB94 (10uM) was added for 2 days. (**h**) Immunoblot with anti c-FMS and α-tubulin antibodies in whole lysates (n=3). (**i**) Soluble c-FMS was detected by ELISA (n=3). The treatment of BB94 inhibited the shedding of c-FMS and diminished soluble c-FMS in the media. All data are shown as mean ± SEM. *; $p < 0.05$ significant by two-tailed, paired *t*-test (a and **i**). M: mature form, I: immature form, H: hgh mass, L: low mass.

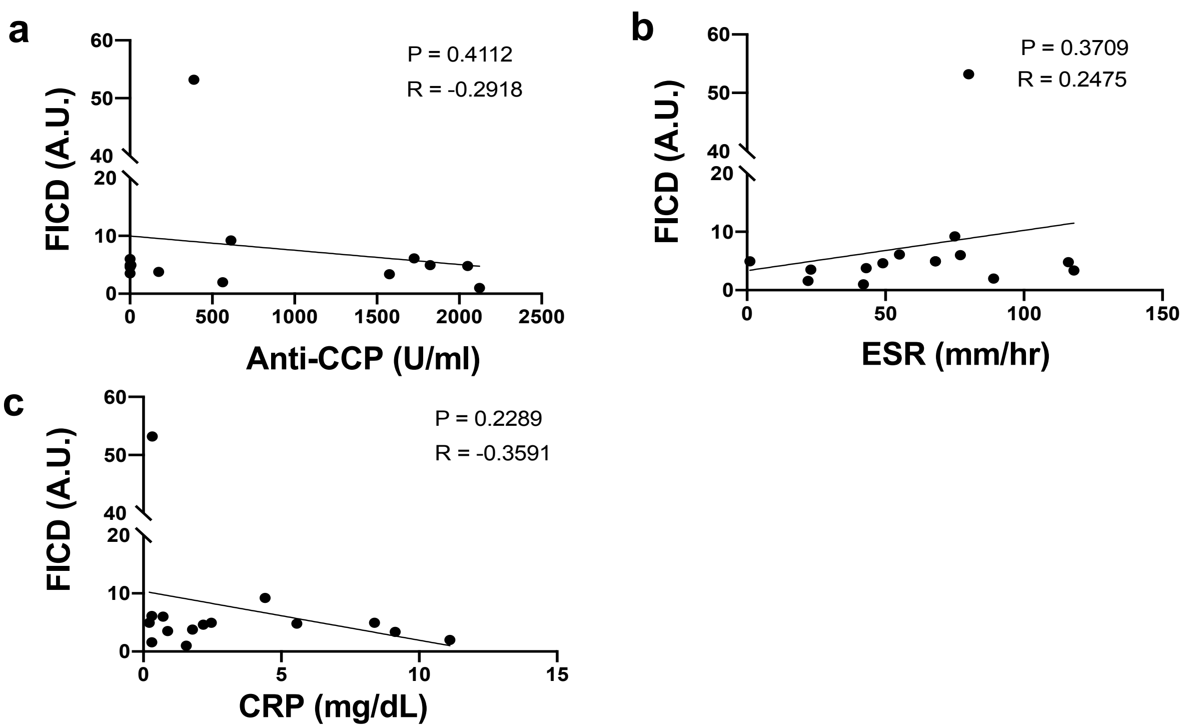

Supplemental Fig. 2. The correlation between the expression of FICDs and serum factors. Synovial fluid and serum were collected from patients with inflammatory rheumatoid arthritis (RA). Synovial CD14+ macrophages were purified by positive selection using anti-CD14 magnetic beads from synovial fluid mononuclear cells, and the expression of FICDs was measured by immunoblot. A correlation plot between FICD expression and anti-cyclic citrullinated peptide (CCP) antibody (**a**), erythrocyte sedimentation rate (ESR, **b**), or C-reactive protein, (CRP, **c**) are shown.

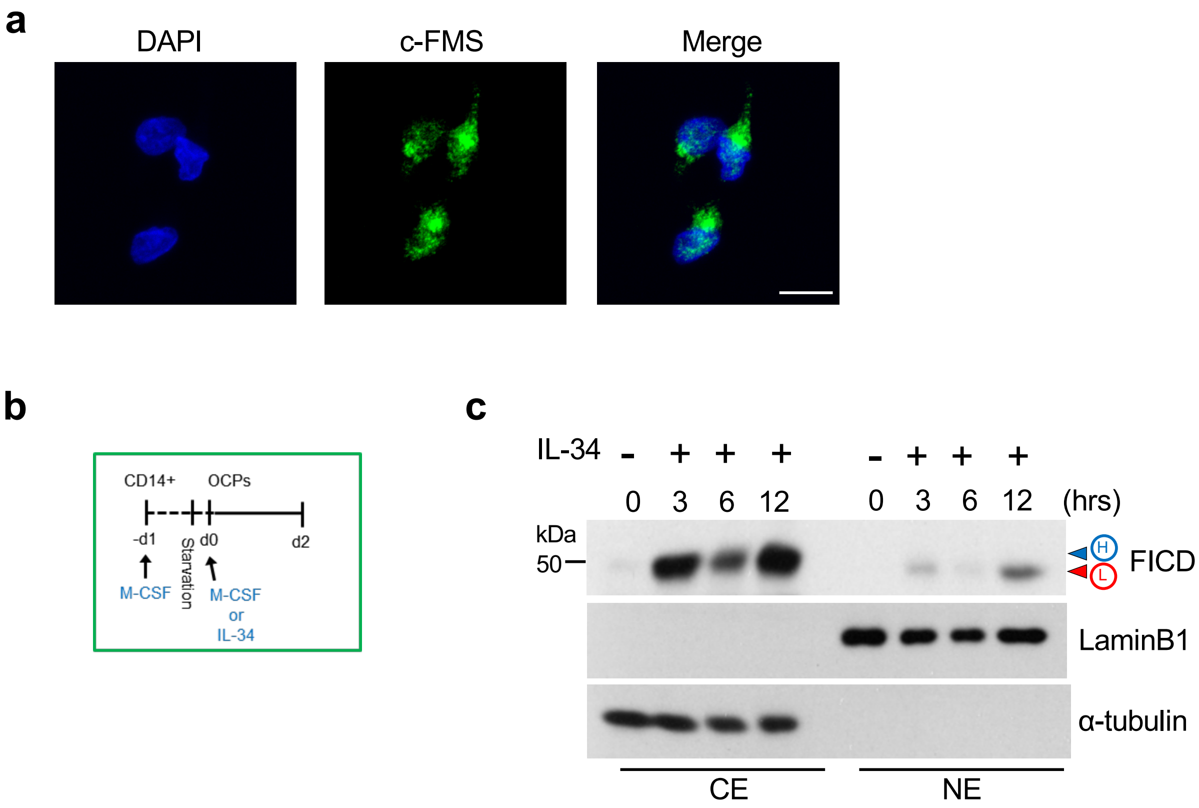

Supplemental Fig. 3. The cellular localization of FICDs.
(**a**) Confocal microscopy of human CD14+ cells labeled with antibodies against C-terminal of c-FMS (middle) and DAPI (Left). White scale bar is 10 μm. (**b** and **c**) Human CD14+ cells were cultured with M-CSF for 12hrs and then was stimulated with IL-34 (20 ng/ml) for the indicated time (**c**). (**b**) A schematic diagram illustrating the experiment design for Supplemental Fig. 2**c** and Fig. 2**c**, (**c**) Immuno-blot of cytoplasmic and nuclear lysate with anti c-FMS, Lamin B1, and α-tubulin antibodies. M: mature c-FMS, I: immature c-FMS, H-FICD: high mass FICD, L-FICD; low mass FICD. Data represent at least three independent experiments.

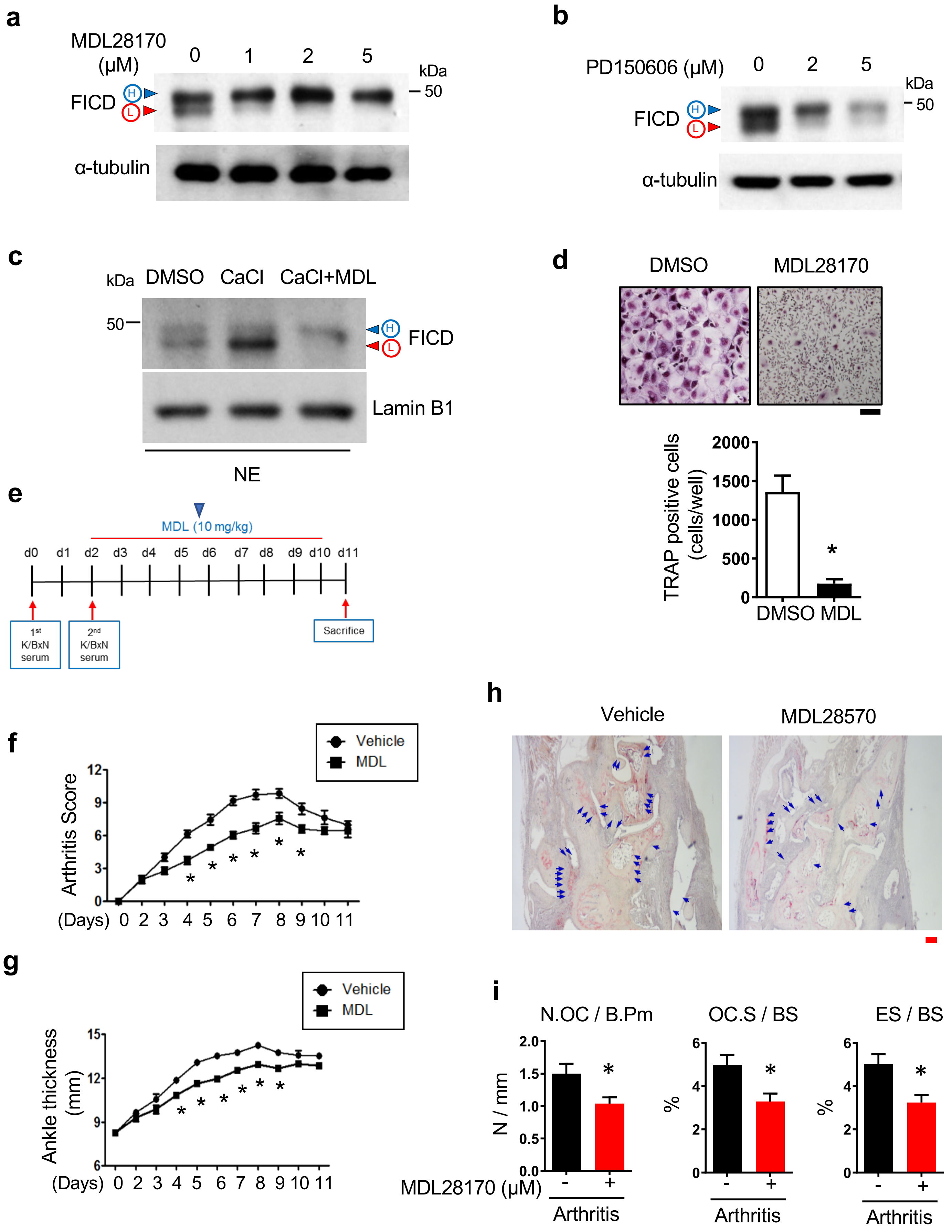
a
MDL28170 (μM)
0 1 2 5
kDa
50
FICD
H
L
α-tubulin
b
PD150606 (μM)
0 2 5
kDa
50
FICD
H
L
α-tubulin
c
kDa
DMSO CaCl CaCl+MDL
50
FICD
H
L
Lamin B1
NE
d
DMSO MDL28170
TRAP positive cells (cells/well)
2000
1500
1000
500
0
*
DMSO MDL
e
MDL (10 mg/kg)
d0 d1 d2 d3 d4 d5 d6 d7 d8 d9 d10 d11
1st K/BxN serum
2nd K/BxN serum
Sacrifice
f
Vehicle
MDL
Arthritis Score
12
9
6
3
0
(Days) 0 2 3 4 5 6 7 8 9 10 11
g
Vehicle
MDL
Ankle thickness (mm)
15
12
9
6
(Days) 0 2 3 4 5 6 7 8 9 10 11
h
Vehicle
MDL28570
i
N.OC / B.Pm
OC.S / BS
ES / BS
N / mm
2.0
1.5
1.0
0.5
0.0
%
6
4
2
0
MDL28170 (μM)
- +
Arthritis

Supplemental Fig. 4. Calpain regulates FICD generation.

(**a, b**) Human CD14+ cells were cultured with M-CSF (20 ng/ml) for 8 hours to induce early signals and then (**a**) MDL 28170(0, 1, 2, 5uM) or (**b**) PD150606 (0, 2, 5 uM) was added for 2 days. Immunoblot with anti c-FMS and α-tubulin antibodies in whole lysates(n=3). (**c**) Immunoblot of nuclear lysates with c-FMS and Lamin B1 antibodies. Cells were cultured with M-CSF for 12 hours, and then M-CSF was removed**.** Cells were subsequently treated with or without $CaCl_2$ (10 mM) for an additional 24 hours. (**d**) Osteoclastogenesis assay. Human CD14+ cells were cultured with M-CSF, RANKL and MDL28170 for 4 days. Upper panel shows representative images of TRAP-stained cells. Lower panel shows the percentage of TRAP-positive multinuclear cells (MNCs: more than three nuclei) per control (DMSO) from three independent experiments. Black scale bar is 100 µm. . (**e** - **i**) K/BxN serum transfer induced arthritis model. 8-week old male C57BL/6J mice were received K/BxN serum on day 0 and day 2. Vehicle or MDL28170 (10 mg/kg) was administrated intraperitoneally (i.p) at day 2 until day 11 (n=10). (**e**) K/BxN Experimental design. (**f**) Arthritis score. (**g**) Ankle thickness. (**h**) Representative images of TRAP stained histological sections from calcaneocuboid and tarsometatarsal joints. (**i**) Histomorphometry analysis of tarsal bones. N.OC / B.Pm; Osteoclast number / bone parameter. OC.S / BS; osteoclast surface / bone surface. ES / BS; Eroded surface / bone surface (n=7). All data are shown as mean ± SEM. *, $p < 0.05$ by two-tailed, unpaired *t*-test (**d**,**i**) or One-way ANOVA with a post hoc Tukey test (**f**,**g**).

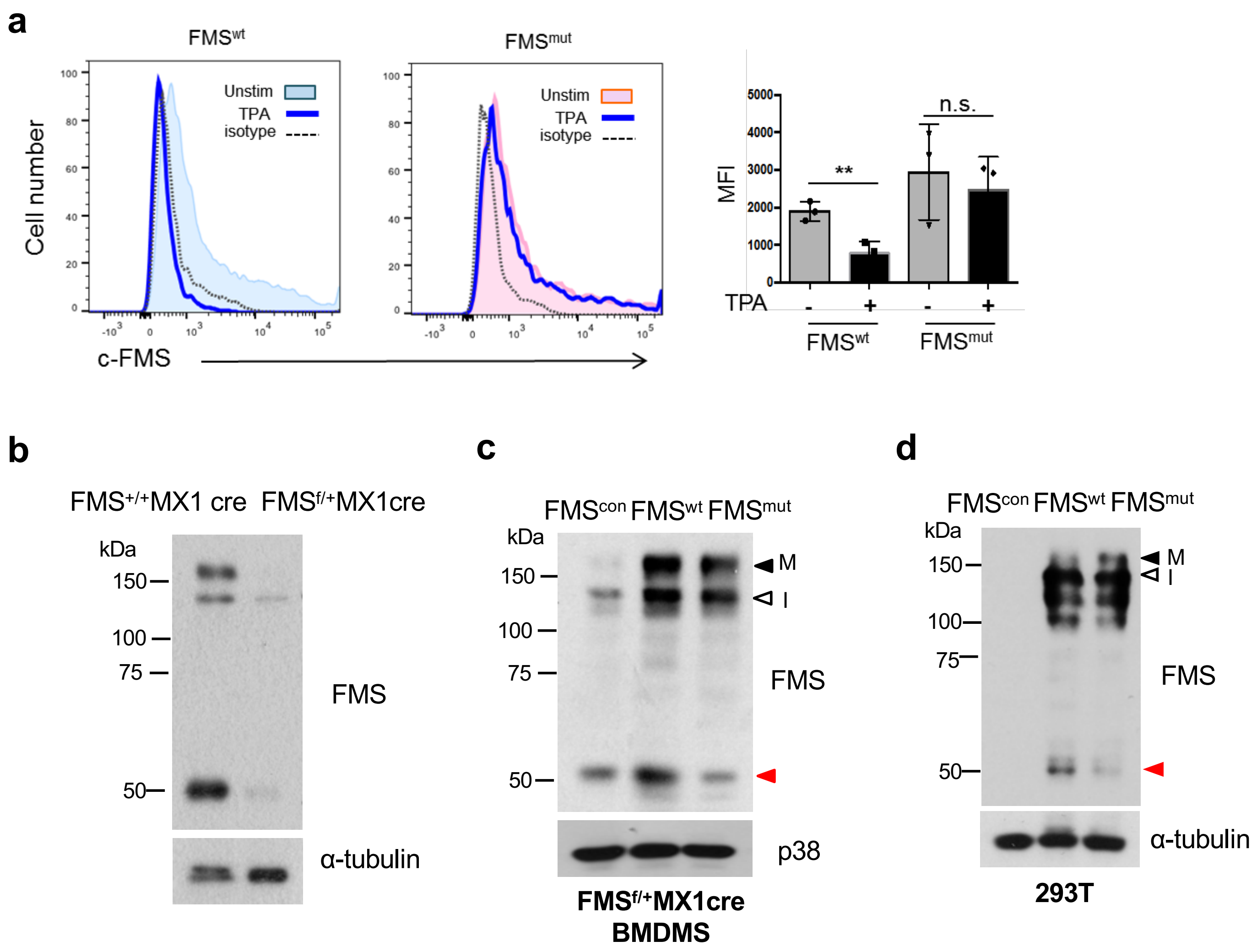

Supplemental Fig. 5. c-FMS proteolysis and TACE-cleavage resistant form of c-FMS.
(**a**) 293T cells has no c-FMS expression and were transduced by lentiviruses encoding control, FMS^{wt}, or FMS^{mut} and then stimulated with or without 12-O-Tetradecanoylphorbol-13-acetate (TPA, 100 ng/ml) to activate TACE. The expression of FMS^{wt} and FMS^{mut} was analyzed by flow cytometry. Left panel shows representative images, and right panel shows the accumulative quantification from three independent experiments. (**b**) Protein expression analysis of c-FMS by immunoblot. BMDMs from FMS$^{+/+}$MX1 cre and FMS$^{f/+}$MX1cre mice were cultured with M-CSF (20ng/ml) for two days. The levels of FICD in FMS$^{f/+}$MX1cre BMDMs (lane 2) were 20% of FMS$^{+/+}$MX1cre (lane 1) (**c, d**) Protein expression analysis of FMS^{wt} and FMS^{mut} by immunoblot. FICD expression was diminished in FMS^{mut} compared to FMS^{wt} in BMDMs (**c**) or 293T cells (**d**) Representative results from at least three independent experiments. All data are shown as mean ± SEM. *; $p < 0.05$ **; $p < 0.05$, n.s; not significant by two-tailed, paired *t*-test (**a**). Arrow: FICD. All data represent at least three experiments.

a

Calpain cleavage sequences in c-FMS

Position	Peptide	Score
31	VIEPSVPELV | VKPG	0.764
70	YSDGSSSILS | TNNA	0.829
73	GSSSILSTNN | ATFQ	0.696
224	IRGEAAQIVC | SASS	0.674
304	VVESAYLNLS | SEQN	1.049
383	SEAGRYSFLA | RNPG	0.88
619	VLKVAVKMLK | STAH	0.829
631	AHADEKEALM | SELK	0.78 ◁ FICD
683	FLRRKAEAML | GPSL	0.674
735	MRPVSTSSND | SFSE	0.726
737	PVSTSSNDSF | SEQD	0.821
918	FLQEQAQEDR | RERD	0.853
924	QEDRRERDYT | NLPS	0.677
933	TNLPSSSRSG | GSGS	0.796
934	NLPSSSRSGG | SGSS	0.948
935	LPSSSRSGGS | GSSS	0.747
936	PSSSRSGGSG | SSSS	1.41
937	SSSRSGGSGS | SSSE	1.038
938	SSRSGGSGSS | SSEL	1.033
939	SRSGGSGSSS | SELE	1.014
943	GSGSSSSELE | EESS	1.06
946	SSSSELEEES | SSEH	0.764

b

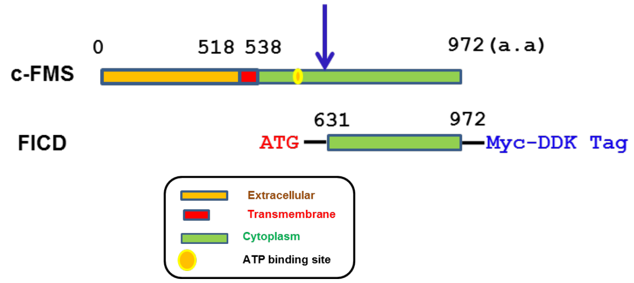

c

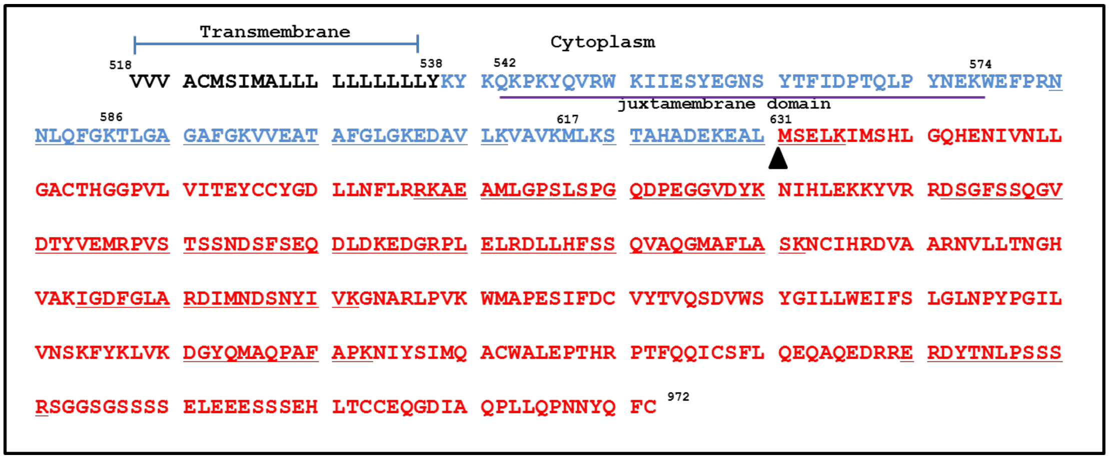

Supplemental Fig. 6. Calpain regulates FICD generation.
(**a**) A table showing the calpain cleavage site in c-FMS predicted by GPS-CCD program. (**b**) A schematic diagram of a FICD construct. A predicted calpain cleavage site (black arrow) in cytoplasmic domains of c-FMS was used for a FICD construct and MYC-HA double tag was added into c-terminal regions of a FICD construct. (**c**) A sequence of a FICD construct in red.

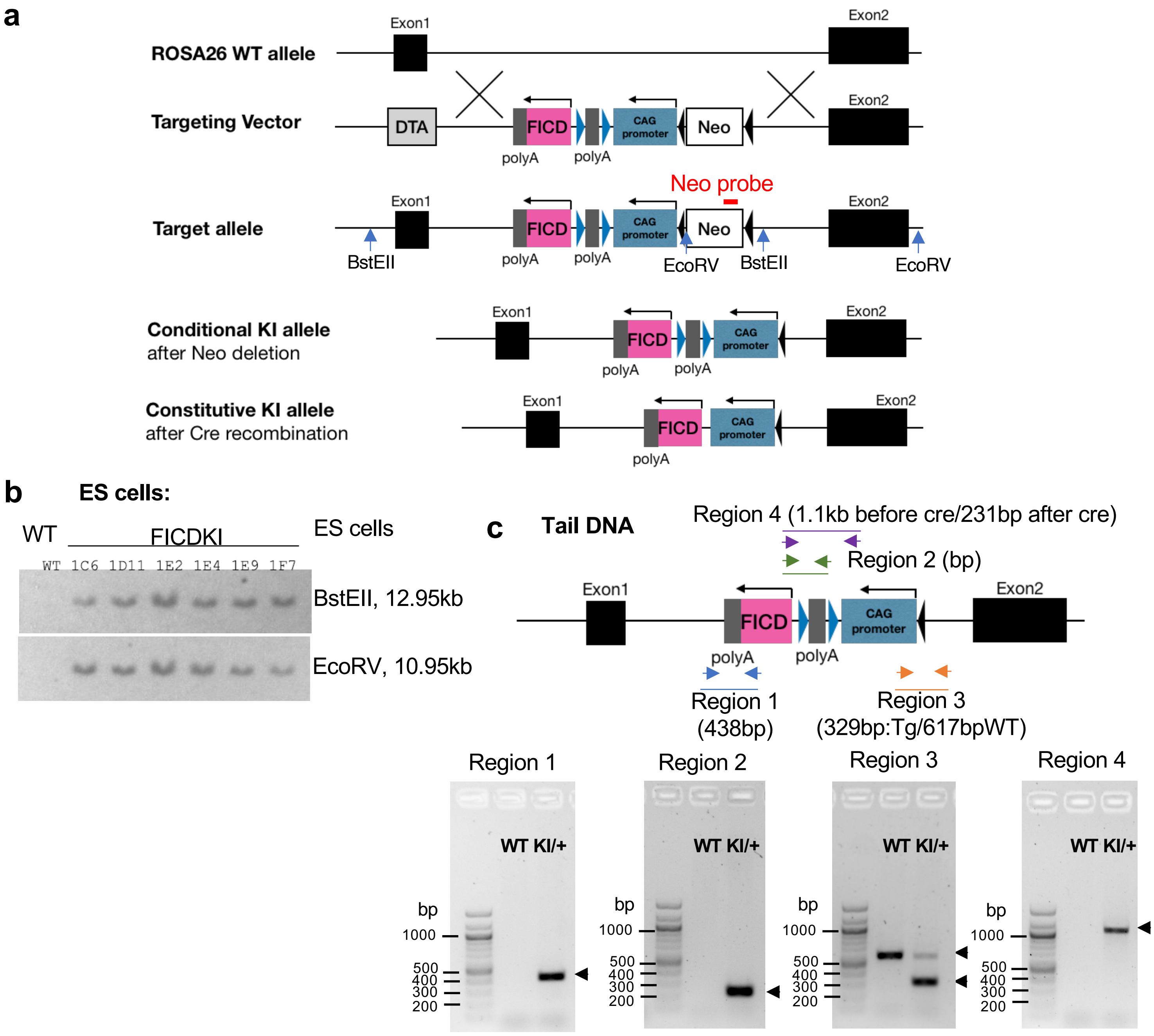

Supplemental Fig. 7. Generation of FICDKI/KI^{M} mice.
(**a**) Construction of the FICD-HA knock-in (KI) targeting vector. See the Experimental Procedures for details. The gene encoding ROSA26 (WT allele); the targeting vector (targeting vector); poly A (filled gray square); loxP sequence (filled blue triangle); SDA (self-deletion anchor) site (filled black triangle); CAG promoter; Neo cassette; Diphtoxin A gene (DTA); the targeted allele with the targeting vector (target allele); SDA mediated Neo deletion (Conditional KI allele); cre-mediated expression after removal of polyA between CAG promoter and FICD-HA gene (Constitutive KI allele). (**b**) Southern blot analyses of DNA from WT ES cells or FICDKI ES cells. Genomic DNA was extracted from ES cells, digested with BstEII or EcoRV, and analyzed by Southern blot by Neo probe (red) shown in Figure A. Southern analysis with Neo probe generates 12.95kb fragments after digestion with EstEII (upper panel) and 10.95kb fragments after digestion with EcoRV (lower panel). (**c**) PCR analyses of DNA from wild type LysM cre (WT), FICD$^{KI/+}$ LysM cre or FICD$^{KI/KI}$ LysM cre mice. Genomic DNA was extracted from mouse tail tissue. Primers for PCR are shown as arrows. The sequence of primers is listed in Table S2.

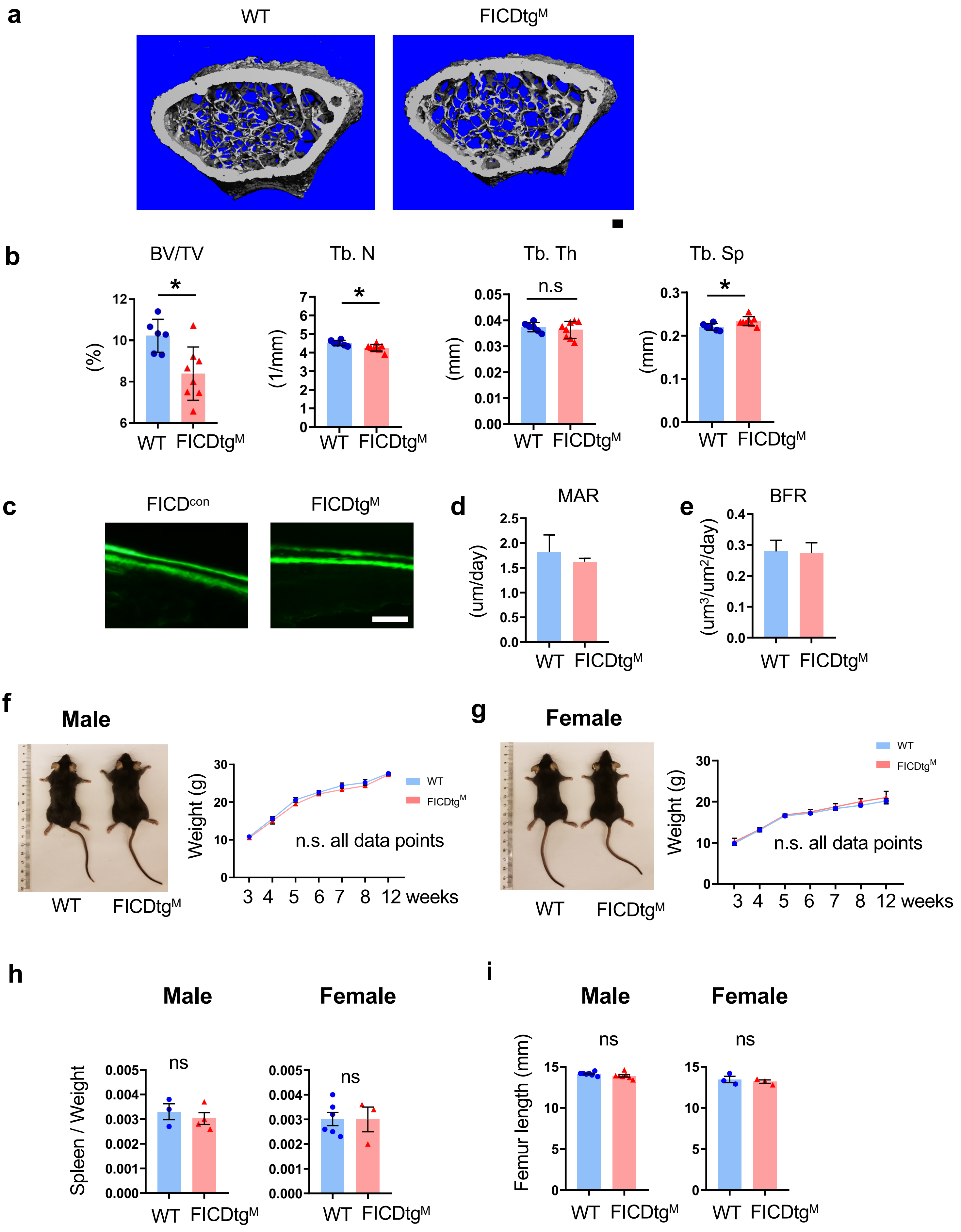
a
WT
FICDtgM
b
BV/TV
*
(%)
WT FICDtgM
Tb. N
*
(1/mm)
Tb. Th
n.s
(mm)
Tb. Sp
*
(mm)
c
FICDcon
FICDtgM
d
MAR
(um/day)
e
BFR
(um3/um2/day)
f
Male
WT FICDtgM
Weight (g)
n.s. all data points
3 4 5 6 7 8 12 weeks
g
Female
h
Male
Female
Spleen / Weight
ns
i
Femur length (mm)
ns

Supplemental Fig. 8. Overt phenotype of FICDtg^{M} mice is comparable to wild type.
(**a, b**) μ-CT analysis of femurs from 12-week-old female wild type (WT, n=6) and FICDtg^{M} mice (n=7). (**a**) Representative images. (**b**) Bone parameters in distal femurs. Bone volume/tissue volume ratio (BV/TV), trabecular thickness (Tb.Th), trabecular numbers (Tb.N), and trabecular space (Tb.Sp) were determined by μ-CT analysis. Black scale bar: 100 μm. (**c** – **e**) Bone formation parameters of 12 weeks old male WT(n=5) and FICDtg^{M} (n=7) mice. Representative images of calcein double labeling in the trabecular bone (**c**), White scale bars: 10 μm. (**d**) Mineral apposition rate (MAR) and (**e**) Bone formation rate (BFR).The comparison of body weight between wild type (WT) and FICD^{tgM} male (**f**) and female (**g**) mice. (**h**)The comparison of spleen weight between wild type (WT) and FICDtg^{M} male and female mice. (**i**) The comparision of femur length between wild type (WT) and FICDtg^{M} male and female mice. All data are shown as mean ± SEM. ns, not significant; *, $p < 0.05$ by two-tailed, unpaired *t*-test (**b**, **d**, **h**, **i**) or One-way ANOVA with *a post hoc Tukey test* (**f, g**) .

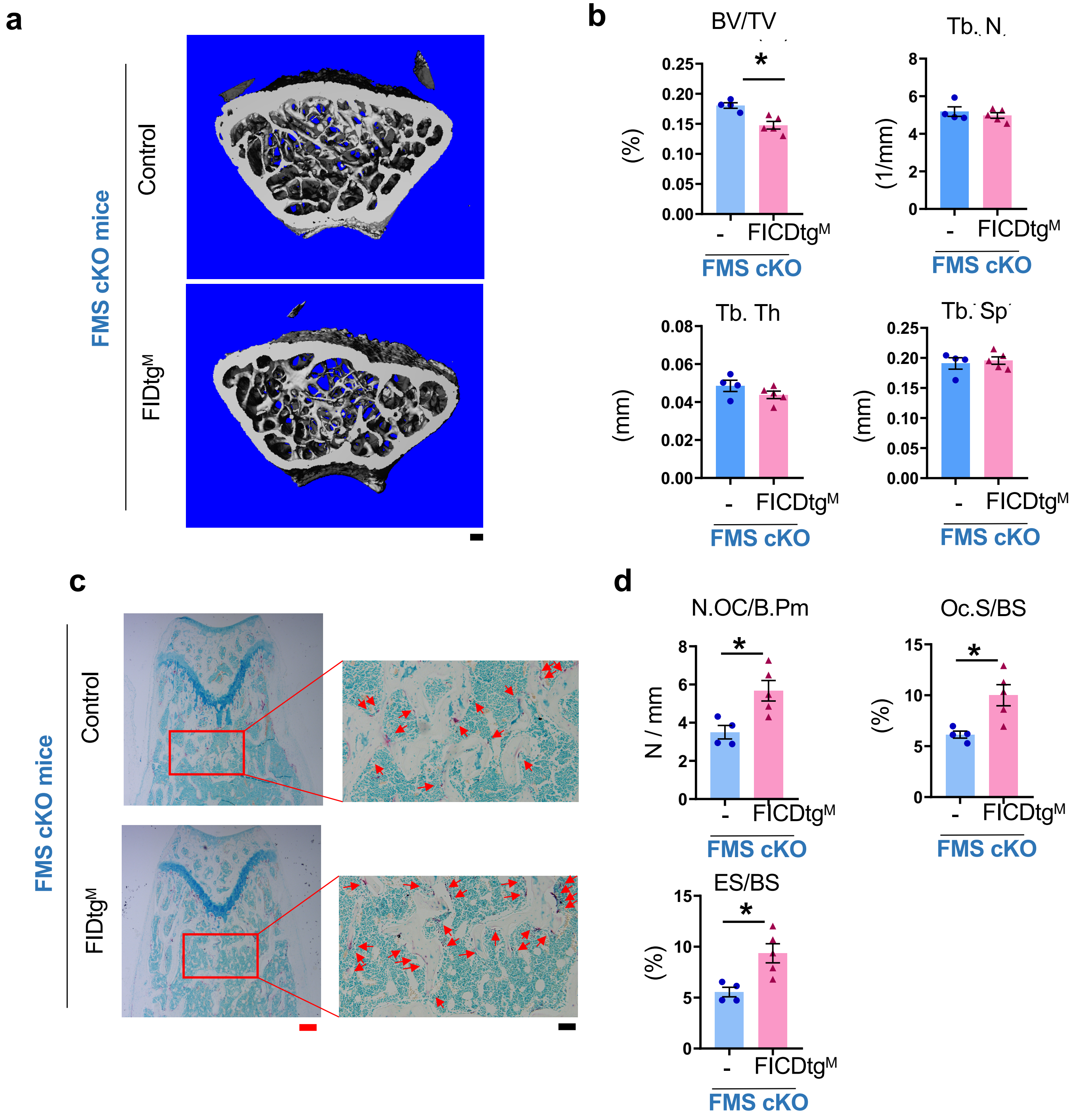

Supplemental Fig. 9. FICD expression in FMS null background diminished bone mass and increased osteoclast numbers.

(**a, b**) μ-CT analysis of femurs from 12-week-old male FMS cKO(control, n=6) and FMScKOFICDtg^{M} mice (n=7). (**a**) Representative images. (**b**) Bone parameters in distal femurs. Bone volume/tissue volume ratio (BV/TV), trabecular thickness (Tb.Th), trabecular numbers (Tb.N), and trabecular space (Tb.Sp) were determined by μ-CT analysis. (**c, d**) Histomorphometry analysis of the distal femur of 12-week-old male FMS cKO(control, n=4) and FMScKOFICDtg^{M} mice (n=5). (**c**) Representative images showing TRAP-positive, multinucleated osteoclasts (red arrow). (**d**) The number of osteoclasts per bone surface (N.Oc/BS), osteoclast surface area per bone surface (Oc.S/BS), and eroded surface per bone surface (ES/BS). Black scale bar is 100 μm and red scale bar is 200 μm. All data are shown as mean ± SEM. *, $p < 0.05$ by two-tailed, unpaired *t*-test (**b, d**).

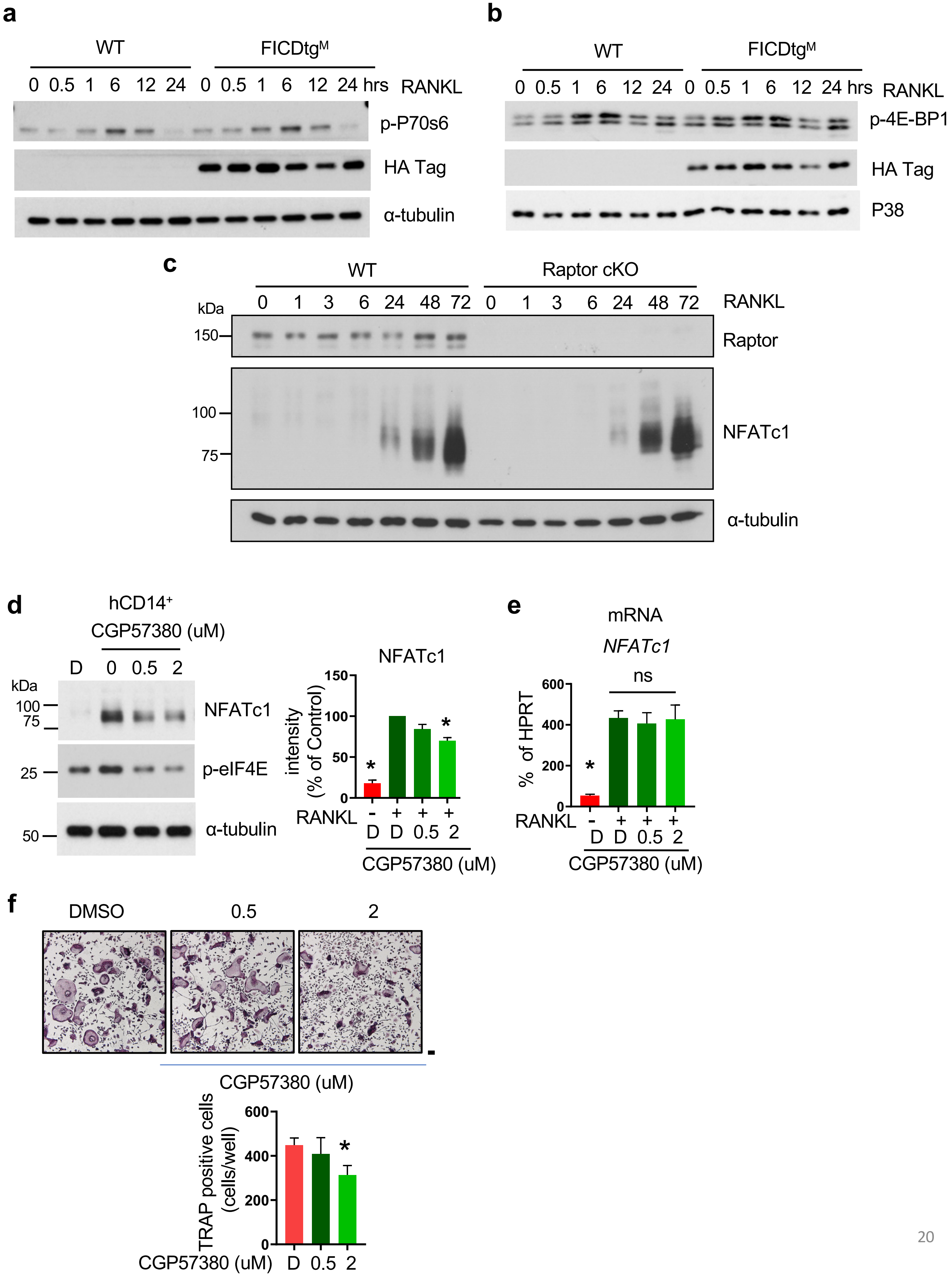

a
WT
FICDtgM
0 0.5 1 6 12 24 0 0.5 1 6 12 24 hrs RANKL
p-P70s6
HA Tag
α-tubulin
b
WT
FICDtgM
0 0.5 1 6 12 24 0 0.5 1 6 12 24 hrs RANKL
p-4E-BP1
HA Tag
P38
c
WT
Raptor cKO
0 1 3 6 24 48 72 0 1 3 6 24 48 72 RANKL
kDa
150
100
75
Raptor
NFATc1
α-tubulin
d
hCD14+
CGP57380 (uM)
D 0 0.5 2
kDa
100
75
25
50
NFATc1
p-eIF4E
α-tubulin
NFATc1
intensity (% of Control)
150
100
50
0
RANKL
- + + +
D D 0.5 2
CGP57380 (uM)
e
mRNA
NFATc1
ns
% of HPRT
600
400
200
0
RANKL
- + + +
D D 0.5 2
CGP57380 (uM)
f
DMSO
0.5
2
CGP57380 (uM)
TRAP positive cells (cells/well)
600
400
200
0
CGP57380 (uM)
D 0.5 2

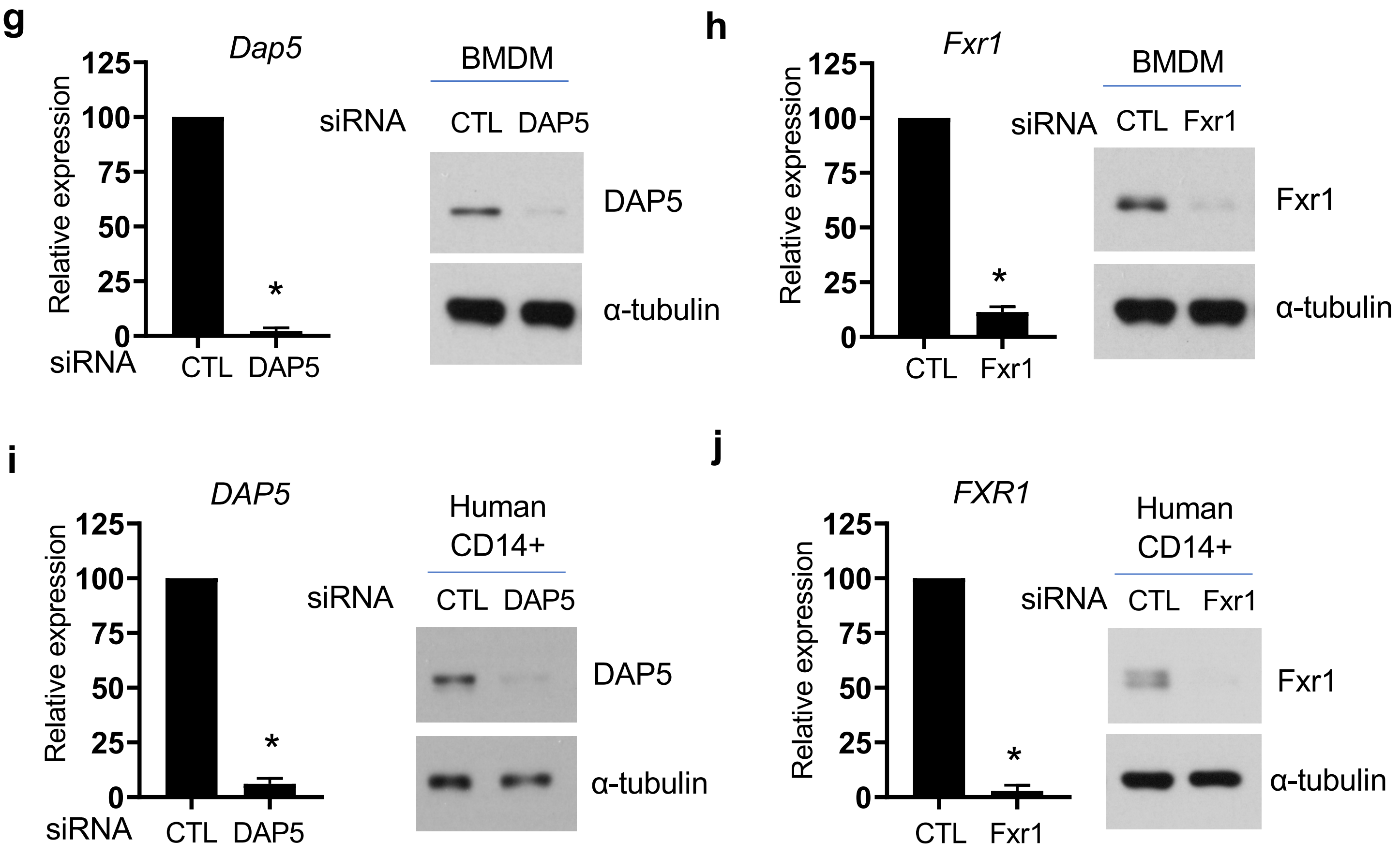

Supplemental Fig. 10. Ablation of Raptor has a minimal effect on NFATc1 protein expression. (**a** and **b**) BMDMs from WT and FICDtg^{M} mice were cultured with RANKL for 24 hours. Immunoblot of whole cell lysates with phopho-p70S6K and phopho-4E-BP1 antibodies. HA-tagged FICDs were detected by HA-antibody. α-tubulin or P38 was used as a control. (**c**) BMDMs from female wild type LysM cre mice (WT) or RAPTOR$^{f/f}$-LysM cre mice (Raptor cKO) were cultured with M-CSF for 4 days and were stimulated with RANKL for the indicated days. Immunoblot of whole cell lysates with anti-Raptor, NFATc1, or α-tubulin antibodies. (**d** - **f**) human CD14+ cells treated with CPG57380 at the indicated doses and then cultured with RANKL for one day. (**d**) Immunoblot of whole lysate with anti-NFATc1 antibody. α-tubulin was used as a control. Left panel shows the representative images. Right panel shows the percentage of intensity of NFATc1 bands (24h). The intensity of NFATc1 bands in RANKL treatment conditions (control) was set as 100%. (n = 4) (**e**) Expression mRNA level of NFATc1. (**f**) Osteoclastogenesis assay. BMDMs from WT mice were treated with CPG57380 at the indicated doses and then cultured with RANKL for 3 days. Upper panel shows representative images of TRAP-stained cells. Lower panel shows the percentage of TRAP-positive multinuclear cells (MNCs: more than three nuclei) per control from three independent experiments. Black scale bar: 100 μm. D: DMSO. (**g** - **j**) BMDM (**g**,**h**) and Human CD14^{+} (**I**, **j**) cells were transfected with control (CTL), DAP5, or Fxr1 siRNAs. Respectively, the efficiency of knock-down was measured by qPCR (left panel) and immunoblot with anti-DAP5, Fxr1, or α-tubulin antibodies (right panel). All data are shown as mean ± SEM. *, $p < 0.05$ by one-way ANOVA with *a post hoc Tukey* test (**d**-**f**) or two-tailed, unpaired *t*-test (**g**-**j**). Data represent at least three experiments(**a,b**,**d**-**j**) and 2 biological replicates (**c**).

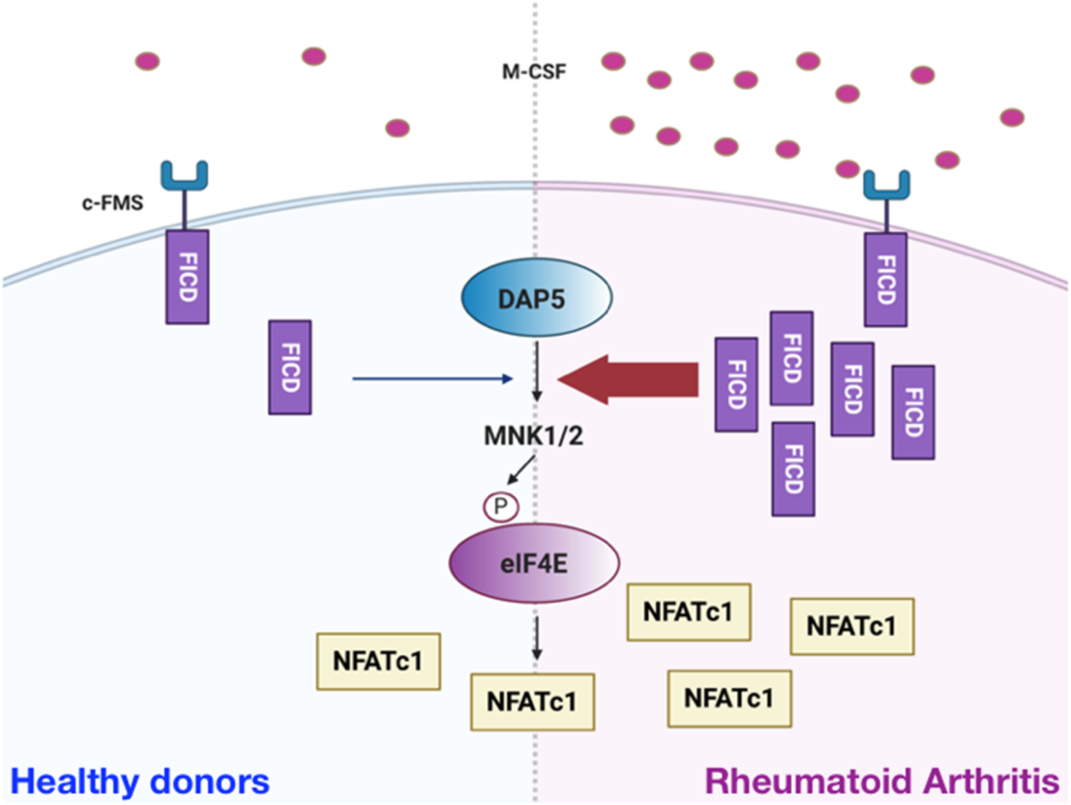

Supplemental Fig. 11. Proposed model: c-FMS proteolysis cooperates with MNK1/2 pathways to promote RANKL-induced osteoclastogenesis.
Under homeostatic conditions c-FMS proteolysis is initiated by the engagement of M-CSF to c-FMS. Small fragments (called FICD) are generated by c-FMS proteolysis at a later phase of c-FMS activation. FICD forms a complex with DAP5 (eIF4G2) and its activity is integrated with MNK1/2 to promote eIF4E activation. Upon stimulation with RANKL, FICD interacts with protein translation and gene expression pathway proteins to drive NFATc1 protein expression and osteoclastogenesis. Under high-MCSF conditions, such as rheumatoid arthritis synovium, constitutive c-FMS signaling augments and maintains FICD generation and thus promote osteoclast differentiation. Therefore, our findings suggest that c-FMS proteolysis may be differentially regulated under inflammatory and homeostatic conditions to fine-tune osteoclast differentiation and function.

Supplemental table 1. FICD interacting Proteins

CSF1R	PDHB	TBL2	SPCS3	TAF8
NUP133	PSMC3	UFL1	TAF5L	THOC5
NUP107	PSMC5	XPNPEP3	YARS2	VAPB
NKRF	UFD1	ARF4	AAR2	ABCF1
NDUFS1	ATP5O	BTAF1	ANAPC1	ATP5J2
NUP98	GTF3C3	CBX8	CCDC47	AURKB
PSMD2	U2SURP	DLAT	CLPB	BZW1
MOGS	ATP5C1	HLTF	CTNNBL1	CDK1
NDUFS2	BPTF	MRPL13	DCUN1D5	CSNK1A1
ERCC3	DPM1	NUP43	DLST	EIF3F
LRPPRC	DRG1	POLR1A	EIF3H	ERLIN2
ATP5A1	EIF4G2	PRPF38A	GADD45GIP1	EXOSC3
AAAS	KPNA2	SDCBP	GAPDH	FXR1
DDX18	LMAN2	SUPV3L1	GATAD2B	GPD2
GTF2H4	MTA1	TRIP13	GNAI3	HARS
NUP155	NEFL	ARL1	GTF3C2	KPNA5
DDX1	PDS5B	DDX6	HSD17B10	LEMD3
IQGAP1	PSMD6	EIF3L	KIF11	LGALS3BP
MTA2	STRAP	EMC3	KPNA3	MCCC2
PSMD1	TUBGCP2	MRPL3	LUC7L3	MRPL44
SMARCD2	XPO5	MRPS31	NDUFS8	MRPL53
DDB1	ANAPC5	MTDH	PELP1	NDUFB4
DNAJA1	CDC73	MTHFD1L	POP1	NGDN
EFTUD2	CFL1	ORC4	PPIE	NRAS
PSMD3	GTF3C5	POLDIP2	PRKAG1	PTRH2
RPN2	HSD17B12	REXO4	PSMB4	RACGAP1
ANAPC4	HYOU1	RFC1	RARS	SRPK1
CUL1	POGZ	RFC3	RPL32	SUZ12
NDUFA10	PPAN	SEC63	SIN3A	TSG101

Supplemental table 2. Primers and Oligonucleotides

A list of primers used in this study

Real-time PCR primers	
Gene Symbol	Sequence
hTACE	Forward: 5'-ACCCTTTCCTGCGCCCCAGA-3'<br>Reverse : 5'-GTTTTGGAGCTGCTGGCGCC-3'
hCAPN1	Forward: 5'-TGCCGTTTGCTGAGTGTCC-3'<br>Reverse : 5'-TCTCCTCCGACATCCTCGGG-3'
hCAPN5	Forward: 5'-CTCGGCCGGTGTTCCC-3'<br>Reverse : 5'-CCGGCGTGCCCTTATAGTAG-3'
hCAPN6	Forward: 5'-GCTGTTCCATTGAGTCTCCCA-3'<br>Reverse : 5'-GGGTTTCTCAGGCGAACCAT-3'
hNFATc1	Forward: 5'-CTTCTTCCAGTATTCCACCTAT-3'<br>Reverse : 5'-TTGCCCTAATTACCTGTTGAAG-3'
hHPRT	Forward: 5'-GACCAGTCAACAGGGGACAT-3'<br>Reverse : 5'-CCTGACCAAGGAAAGCAAAG-3'
hFICD	Forward: 5'-TGTCTACACGGTTCAGAGCG-3'<br>Reverse : 5'-GGGTAGGGATTCAGCCCAAG-3'
mNfatc1	Forward: 5'-CCCGTCACATTCTGGTCCAT-3'<br>Reverse : 5'-TCTCCTCCGACATCCTCGGG-3'
mHprt	Forward: 5'-TCCTCAGACCGCTTTTTGCC-3'<br>Reverse : 5'-CTAATCACGACGCTGGGACT-3'
mTnf-α	Forward: 5'-GTCAGGTTGCCTCTGTCTCA-3'<br>Reverse : 5'-TCAGGGAAGAGTCTGGAAAG-3'
mIl-6	Forward: 5'-AAGCCAGAGTCCTTCAGAGAGA-3'<br>Reverse : 5'-GGAAATTGGGGTAGGAAGGA-3'
Genotype primers	
Gene Symbol	Sequence
hFICD-Region_1	Forward: 5'-GGTGCTTGCCTTTATGCCTTTA-3'<br>Reverse : 5'-TGGCTGCCATGAACAAAGGTT-3'
hFICD-Region_2	Forward: 5'-CAGGTCGCCATAGCAACAGTACTC-3'<br>Reverse : 5'-AGTCGCAGATCTGCAAGCTAATTCC-3'
hFICD-Region_3	Forward : 5'-GGGCCATTTACCGTAAGTTATGTAACG-3'<br>Reverse : 5'-GCCATTTAAGCCATGGGAAGTTAG-3'<br>Forward-1: 5'-TGGACAGAGGAGCCATAACTGCAG-3'
hFICD-Region_4	Forward: 5'-GGTACAGGCTCCCAGAAGGTTGAC-3'<br>Reverse : 5'-CAACGTGCTGGTTATTGTGCTGTCT-3'
Oligonucleotides	
Gene Symbol	Sequence
siRNA Negative control	Dharmacon, Cat#; D-001810-10
siRNA: hTACE	Invitrogen, Cat#; HSS186181
siRNA: hCAPN1	Dharmacon, Cat#; L005799-00-0005
siRNA: hCAPN5	Dharmacon, Cat#; L-009423-00-0005
siRNA: hCAPN6	Dharmacon, Cat#; L-009423-00-0005
siRNA: hEIF4G2	Dharmacon, Cat#; L-011263-00-0005
siRNA: hFXR1	Dharmacon, Cat#; L-012011-00-0005
siRNA: mEif4g2	Dharmacon, Cat#; L-064521-00-0005
siRNA: mFxr1	Dharmacon, Cat#; L-045530-00-0005
